# Efficacy of combined immunotherapy and targeted therapy in overcoming barriers to postoperative recurrence in squamous subtype anaplastic thyroid carcinoma with abscess: a case report and literature review

**DOI:** 10.3389/fonc.2025.1477954

**Published:** 2025-03-19

**Authors:** Shuyun Jiang, Xiaowu Wang, Zhijun Ma

**Affiliations:** ^1^ Department of Clinical Medicine, Qinghai University, Xining, Qinghai, China; ^2^ Department of Surgical Oncology, The Affiliated Hospital of Qinghai University, Xining, Qinghai, China

**Keywords:** multitarget kinase inhibitors, immune checkpoint inhibitors, anaplastic thyroid carcinoma, primary squamous cell carcinoma, thyroid abscess

## Abstract

**Background:**

Molecularly targeted therapies and immunotherapy are increasingly being employed in the treatment of aggressive, recurrent thyroid cancer. Evidence from several studies indicates that a significant proportion of tumor patients derive limited benefit from immunotherapy as a monotherapy, with vascular abnormalities in solid tumors contributing to immune evasion. Numerous studies, both domestic and international, have assessed the efficacy of combining immune checkpoint inhibitors with antiangiogenic agents across various tumor types. These studies suggest that such combination therapies are effective in controlling disease progression and extending survival, among other outcomes. Nevertheless, further research is warranted to substantiate these findings and optimize treatment protocols.

**Methods:**

This study aims to describe a patient diagnosed with anaplastic thyroid carcinoma (ATC) combined with primary squamous cell carcinoma of the thyroid (PSCCT) and concurrent thyroid abscess. The patient experienced local recurrence and metastasis following surgical intervention, radiotherapy, and chemotherapy, and was found to be PD-1 negative. Disease progression was effectively controlled through combination therapy with anlotinib and tislelizumab. Additionally, a comprehensive review of the relevant literature was conducted.

**Results:**

The patient exhibited disease recurrence 8 months postoperatively, notwithstanding the administration of adjuvant radiotherapy and chemotherapy. The local recurrent mass demonstrated minimal reduction following 4 cycles of targeted therapy with anlotinib. However, subsequent treatment with a combination of anlotinib and tislelizumab resulted in a substantial reduction of the neck mass and enlarged cervical lymph nodes after 12 cycles. The patient tolerated the combination therapy well, experiencing no significant adverse effects aside from pronounced fatigue. Thus, the combination therapy with anlotinib and tislelizumab proved effective in controlling the disease.

**Conclusion:**

The management of postoperative recurrence of ATC-PSCCT presents significant challenges, as recurrent tumors typically demonstrate increased aggressiveness and resistance to pharmacological interventions, necessitating multimodal therapeutic approaches. Tislelizumab, an immune checkpoint inhibitor, may facilitate immune-mediated tumor clearance through the activation of various immune cells, including natural killer cells and macrophages. Despite the patient’s PD-1 negativity, the combination of anlotinib and tislelizumab may exert synergistic effects through distinct mechanisms, thereby potentially enhancing therapeutic efficacy. The integration of a multi-targeted tyrosine kinase inhibitor within this combination therapy regimen warrants further investigation.

## 1 Introduction

Anaplastic thyroid carcinoma (ATC) constitutes approximately 1% to 2% of all thyroid malignancies (1) and is characterized by its highly aggressive and malignant nature. Thyroid squamous cell carcinoma can be categorized as either primary or secondary. According to the fifth edition of the WHO classification of thyroid tumors published in 2022, thyroid squamous cell carcinoma is classified as a subtype of ATC (2). Both primary squamous cell carcinoma of the thyroid (PSCCT) and ATC exhibit high malignancy. Patients typically present with a prolonged history of thyroid nodules, followed by a rapid enlargement of the neck mass or tumor invasion into adjacent structures such as nerves or the trachea (3–5).

Thyroid abscess is an exceedingly rare infectious condition of the thyroid gland, predominantly observed in patients with congenital pyriform sinus fistulae. Additionally, secondary infections may develop following the liquefaction necrosis of thyroid adenomas and thyroid tumors (6, 7). To date, no cases of ATC concomitant with thyroid abscess have been documented. Herein, we report a case of a patient who presented with a neck abscess and had untreated thyroid nodules for over two decades. The initial diagnosis of thyroid abscess was established through imaging and ultrasound-guided aspiration, with a consideration of potential malignancy. Based on the intraoperative cryosurgery findings, a total thyroidectomy and functional lymph node dissection of the left cervical region were performed. Postoperative histopathological analysis confirmed the diagnosis of ATC-PSCCT in conjunction with Hashimoto’s thyroiditis and a thyroid abscess. Adjuvant radiotherapy was administered post-surgically; however, chemotherapy was discontinued due to grade IV myelosuppression and neurotoxic side effects. The patient received symptomatic treatment and was closely monitored. Despite these interventions, the patient experienced a local recurrence 8 months postoperatively.

Considering the patient’s intolerance to surgery and ongoing radiotherapy, the lesion exhibited minimal reduction following four cycles of anlotinib-targeted therapy. Consequently, a combined regimen of anlotinib and tislelizumab was initiated, leading to a significant reduction in the lesion size after 12 cycles of the combined therapy. The therapeutic efficacy assessment revealed a partial response (PR), effectively controlling disease progression. The patient has been receiving treatment for over 13 months following the relapse, and their overall condition is presently considered satisfactory. This report aims to present a detailed account of the patient’s treatment process, including the post-relapse phase, to provide a reference for the management of similar cases.

## 2 Case presentation

**Figure 1:**
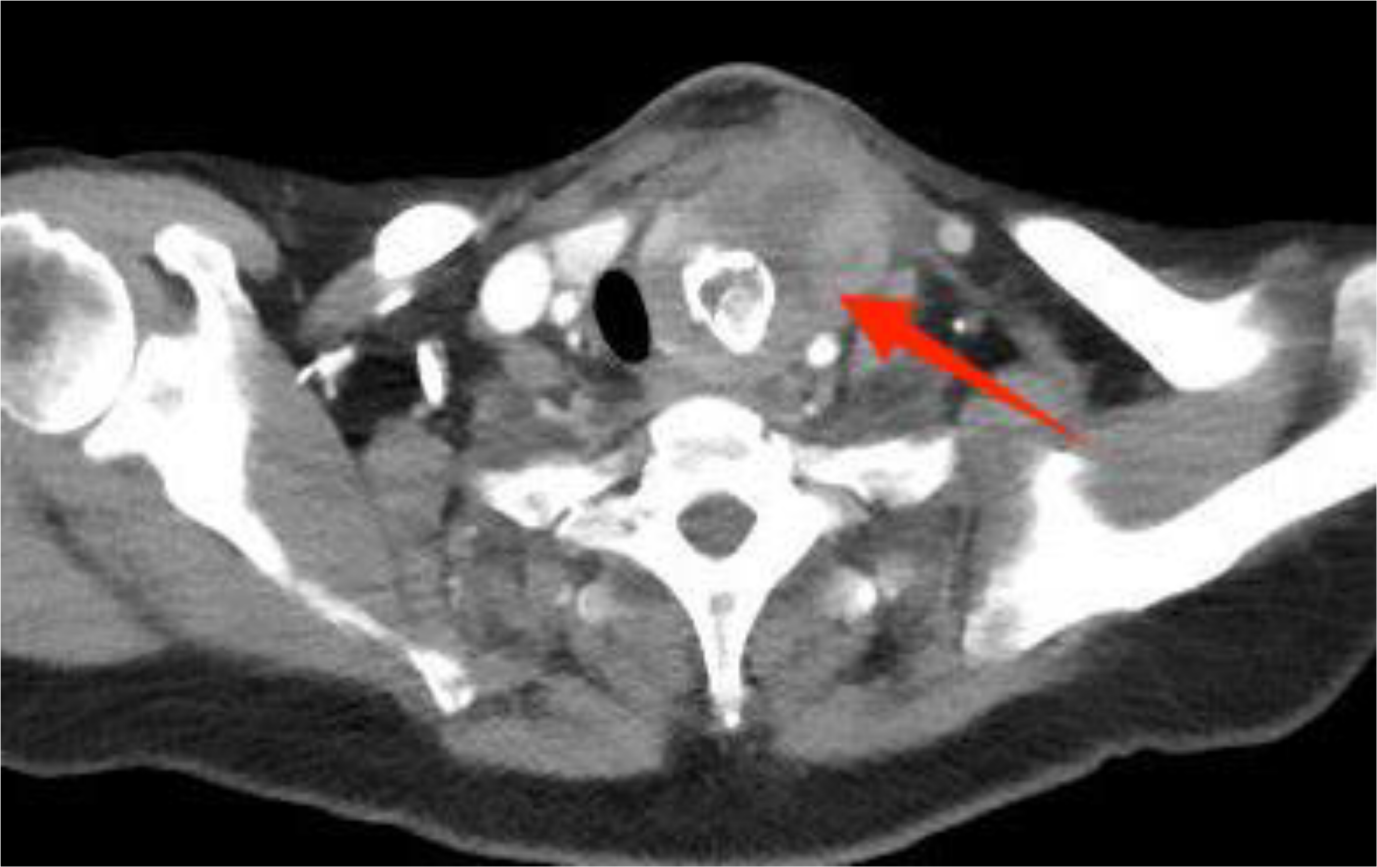
Preoperative contrast-enhanced computed tomography (CT) of the thyroid demonstrated an enlarged left lobe, characterized by internal ring-shaped calcifications and indistinct margins. The trachea was observed to be displaced to the right as a result of compressive forces. Within the mass, areas of low density were identified, suggesting the presence of a lesion, likely indicative of a nodule undergoing liquefaction and necrosis.

**Figure 2:**
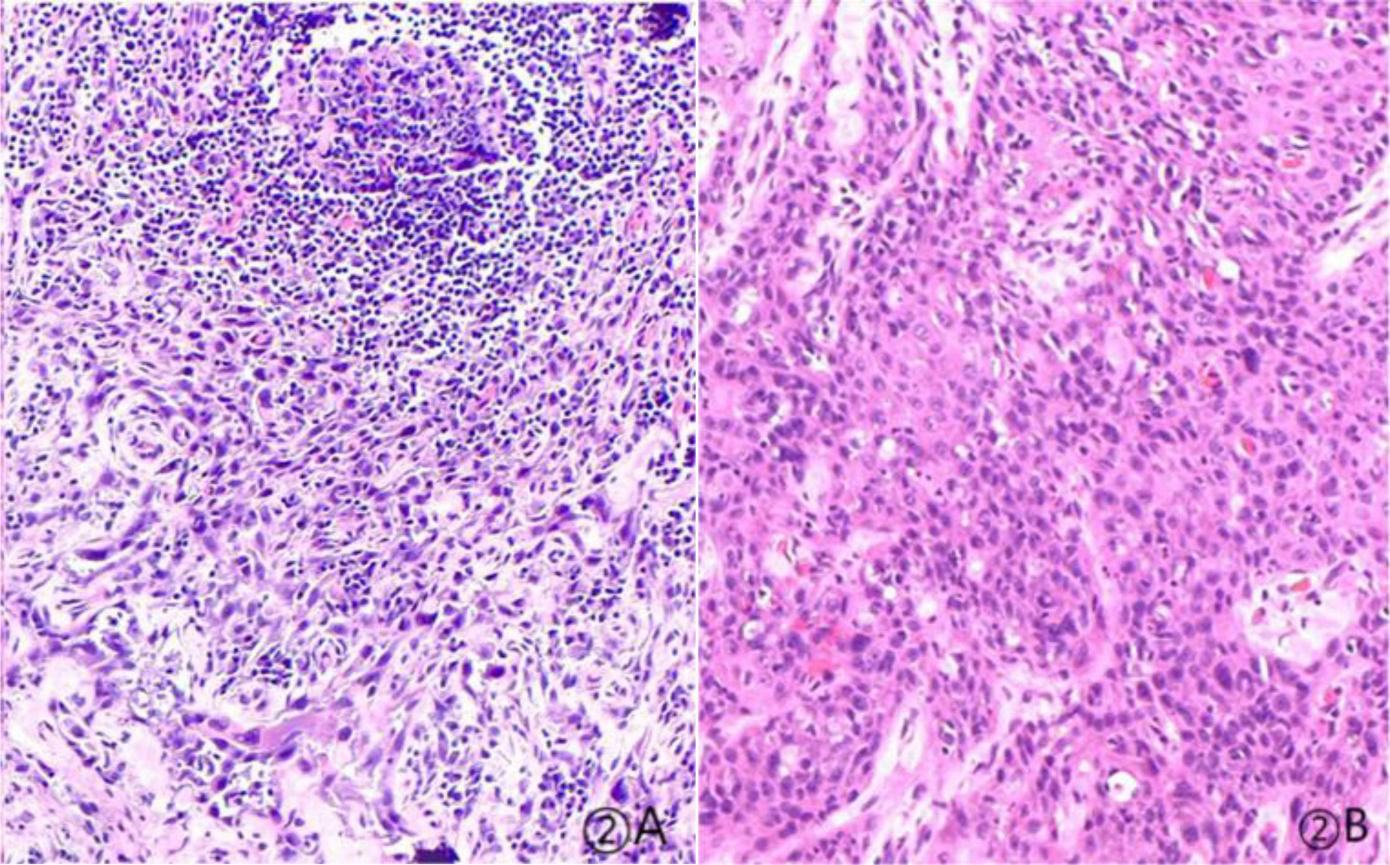
Pathological findings at 20× magnification with Hematoxylin and Eosin (HE) staining: **(A)** Metastasis of squamous cell carcinoma in the cervical lymph nodes, as identified through fine-needle aspiration biopsy (FNAB). **(B)** Tumor cells are organized in solid nests, demonstrating marked cellular heterogeneity.

A 57-year-old woman patient with a 20-year history of thyroid nodules presented with a sudden enlargement of a neck mass, characterized by erythema, swelling, and pain persisting for two weeks. Additionally, she reported experiencing generalized fever in the absence of recent upper respiratory tract infections, neck trauma, hoarseness, or dysphagia. There was no documented history of radiation exposure to the neck, nor was there a family history of thyroid cancer. Physical examination identified erythema, edema, and elevated skin temperature in the left anterior cervical region, accompanied by a 5cm×5cm mass with indistinct borders, restricted mobility, and movement during deglutition. Thyroid ultrasonography revealed a mixed echogenic mass measuring 61mm×51mm×45mm in the left lower pole of the thyroid, categorized as TI-RADS (Thyroid Imaging Reporting and Data System) category 3, with normal morphology observed in the right thyroid lobe. Additionally, the ultrasound detected lymphadenopathy in the left cervical region. CT scan revealed a 5.8cm×5.0cm hypodense mass with ring-shaped calcifications in the left thyroid, compressing the trachea to the right (**Figure 1**). Ultrasound-guided fine needle aspiration biopsy (FNAB) confirmed the presence of necrotic material, neutrophils, and metastatic squamous carcinoma cells in the enlarged lymph nodes(**Figure 2A**). Punctured tissue bacterial culture and gamma-interferon release test were negative. The laboratory results for this patient indicated an elevated leukocyte count (14.70×10^9/L) and neutrophil count (10.76 × 10^9/L). Additionally, there were slight increases in the erythrocyte sedimentation rate (29.00 mm/h), C-reactive protein level (17.90 mg/L), thyroglobulin level (77.12 ng/mL), and thyroglobulin antibody level (55.55 IU/mL). All other test results were within normal limits.

**Figure 3:**
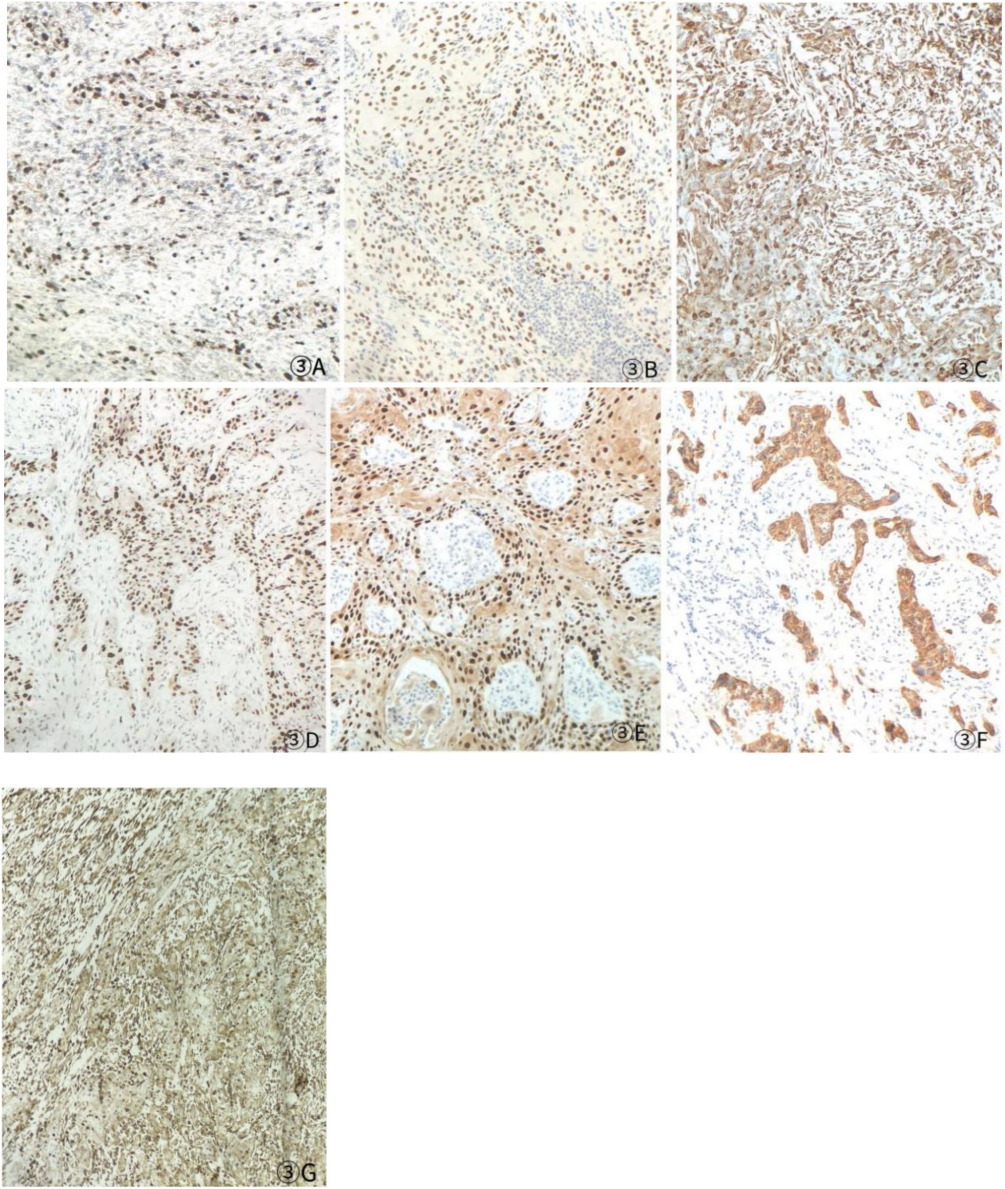
Postoperative immunohistochemistry at 10× magnification: **(A)** Ki-67 (60%), **(B)** TTF-1 (+), **(C)** P40 (+), **(D)** P53 (saturate +), **(E)** P63 (+), **(F)** AE1/AE3 (+), **(G)** Vimentin (Vim) (+).

Based on the patient’s medical history, thyroid function tests, and puncture results, the likelihood of subacute thyroiditis was excluded. Preliminary consideration was given to metastatic cancer in the cervical lymph nodes, left-sided thyroid swelling combined with a thyroid abscess. In view of the fact that the patient had obvious signs and symptoms of infection, but the pathogen had not been identified. Therefore, we empirically used antibiotics combined with local abscess drainage treatment for one week, which resulted in significant improvement of the patient’s infectious symptoms and limitation of the swelling. To further elucidate the origin of squamous epithelial carcinoma cells in the cervical lymph nodes, comprehensive imaging and endoscopic examinations were conducted; however, no primary foci of metastatic carcinoma were identified. A thorough analysis was undertaken to ascertain whether the carcinoma originated from the thyroid gland. Following meticulous preoperative preparation, the patient underwent incision and drainage of a left neck abscess, left thyroid lobectomy, and cryosurgery. Intraoperatively, it was noted that the left thyroid gland was enlarged, the peritoneal membrane of the left lobe was ruptured, the abscess broke down, and a substantial amount of necrotic material was present. After irrigating the operative field, inflammation of the surrounding thyroid tissue was evident, characterized by an indistinct border. Intraoperative frozen section analysis confirmed malignancy in the left thyroid mass, prompting a total thyroidectomy and a left neck functional lymph node dissection. Thyroid tissue and some of the tumor tissue are tightly adherent to surrounding vital tissues and surgery fails to achieve R0 resection. Postoperative pathological examination revealed anaplastic thyroid carcinoma with a squamous cell carcinoma component in the left thyroid, with tumor remnants present at the margins. The squamous cell carcinoma component constituted approximately 50% of the tumor, and an extensive necrotic area was observed, surrounded by changes consistent with Hashimoto’s thyroiditis (**Figure 2B**). Lymph node metastasis of carcinoma was observed in 6 out of 18 nodes. Immunohistochemistry results (**Figure 3**) indicated positive staining for P40, TTF-1, Vimentin, and Ki-67 (60%). Additionally, diffuse positivity for P53, as well as positive staining for P63 and AE1/AE3, were noted. Based on these findings, in conjunction with relevant auxiliary tests, the final diagnosis was determined to be the ATC-PSCCT subtype, accompanied by Hashimoto’s thyroiditis.

After undergoing calcium supplementation and levothyroxine tablet replacement therapy postoperatively, the patient received whole neck radiation therapy directed at the cervical lymph nodes in regions Ib, II, III, IV, V, and VI (50 Gy/25 fractions). Following the completion of radiotherapy, the patient was administered four cycles of cisplatin (30 mg, days 1-4) in combination with albumin-bound paclitaxel (300 mg, days 1-2). However, due to the onset of severe adverse effects, including grade IV myelosuppression, chemotherapy was discontinued after the fourth cycle. The patient underwent only a single baseline assessment at the completion of radiotherapy and did not return for follow-up evaluations as scheduled due to personal reasons. Unfortunately, the patient presented voluntarily with dysphagia more than 8 months after surgery. Subsequent enhanced computed tomography (CT) imaging identified a mass in the left thyroid region, accompanied by enlarged lymph nodes in the left cervical and submandibular areas. Coarse needle aspiration of the thyroid gland, along with immunohistochemical analysis, suggested that the recurrent tumor was an anaplastic thyroid carcinoma with a squamous cell carcinoma component(approximately 50%). Genetic analysis revealed no mutations in the BRAF, TP53, TERT, RET, or NTRK genes. Immunohistochemical evaluation indicated a negative PD-1 status. The Circulating Tumor Cells (CTC) test results, as presented in **Table 1**, indicated the detection of 41 single-cell CTCs. These comprised 20 epithelial, 17 mixed, and 4 mesenchymal cells, with no clusters of other CTC types identified. Upon further assessment, it was determined that complete resection of the mass was currently unfeasible. Additionally, the patient’s Eastern Cooperative Oncology Group (ECOG) performance status score was 3, rendering radiotherapy inappropriate.

**Table 1:** Monitoring circulating tumor cells (CTCs) in patients pre- and post-combination therapy.

	Single Cell CTC	Epithelial CTC	Hybrid CTC	Mesenchymal CTC	Other types of CTC cell clusters
Pre-treatment CTC	41	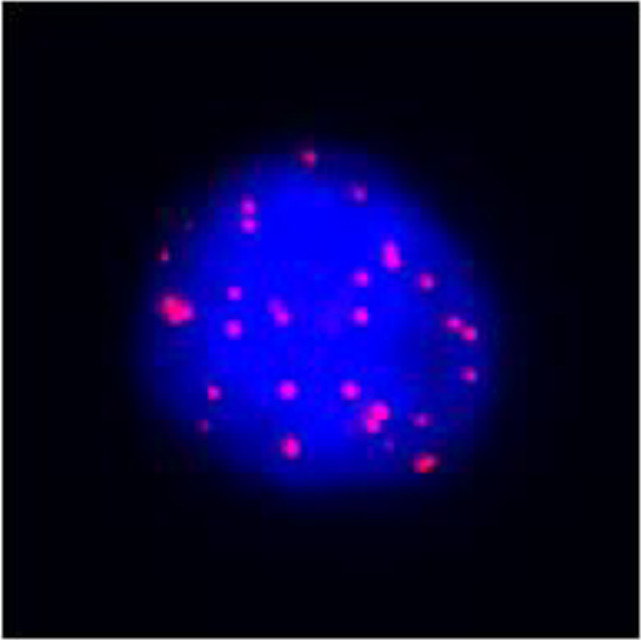 20	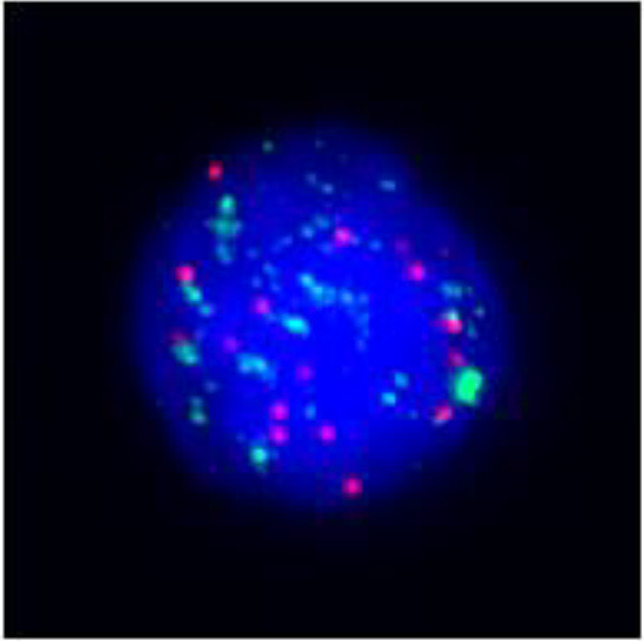 17	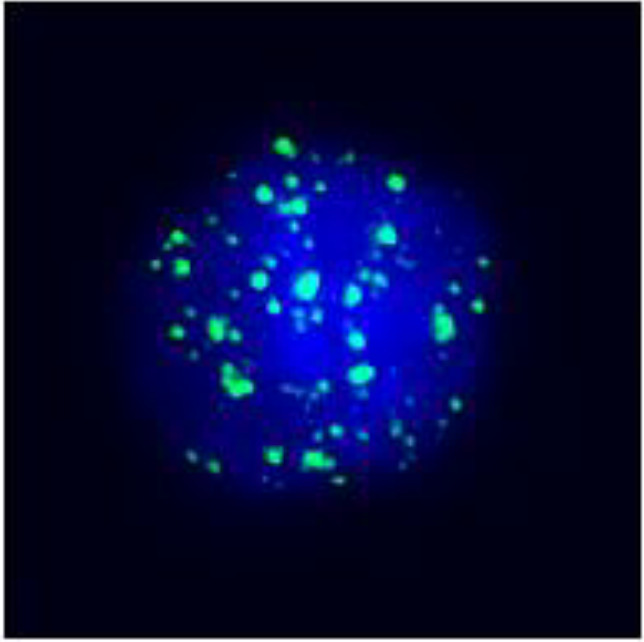 4	0
CTC after 12 cycles of combination therapy	25	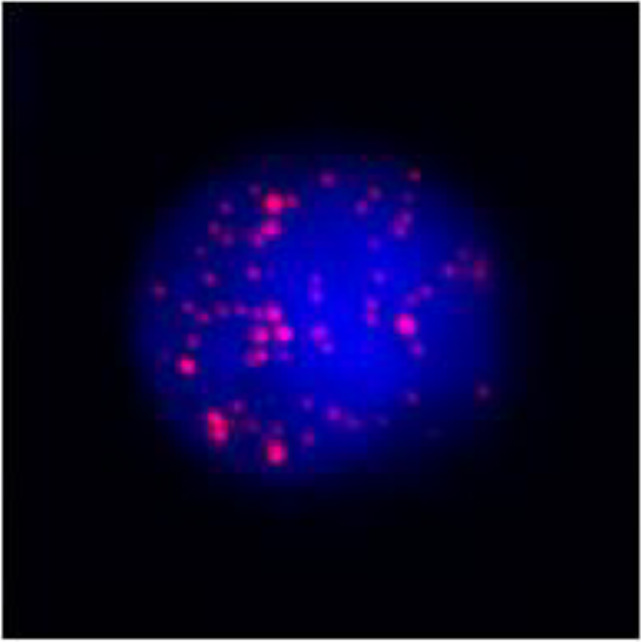 4	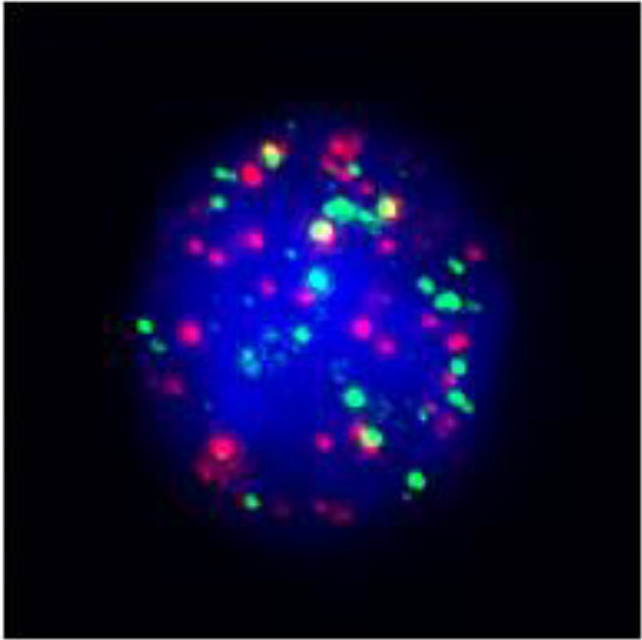 20	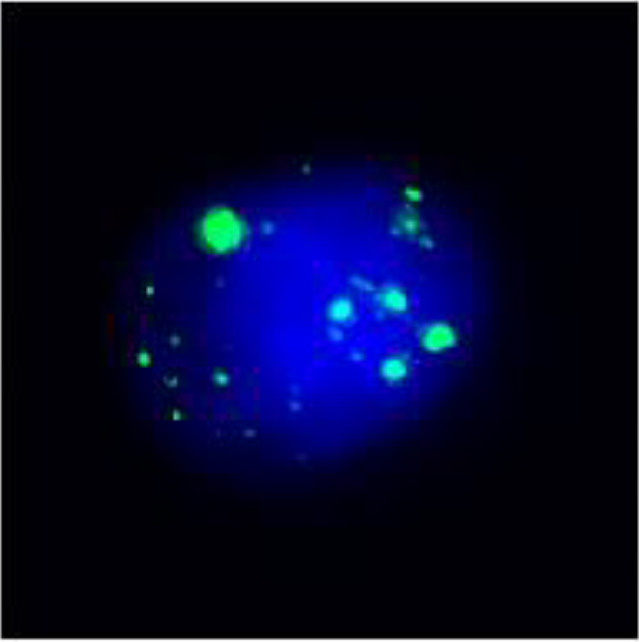 1	0

Epithelial cell markers: CK8, CK18, CK19;Mesenchymal cell markers: TWIST, Vimentin; The staining method used was fluorescence *in situ* hybridization (FISH).

**Figure 4:**
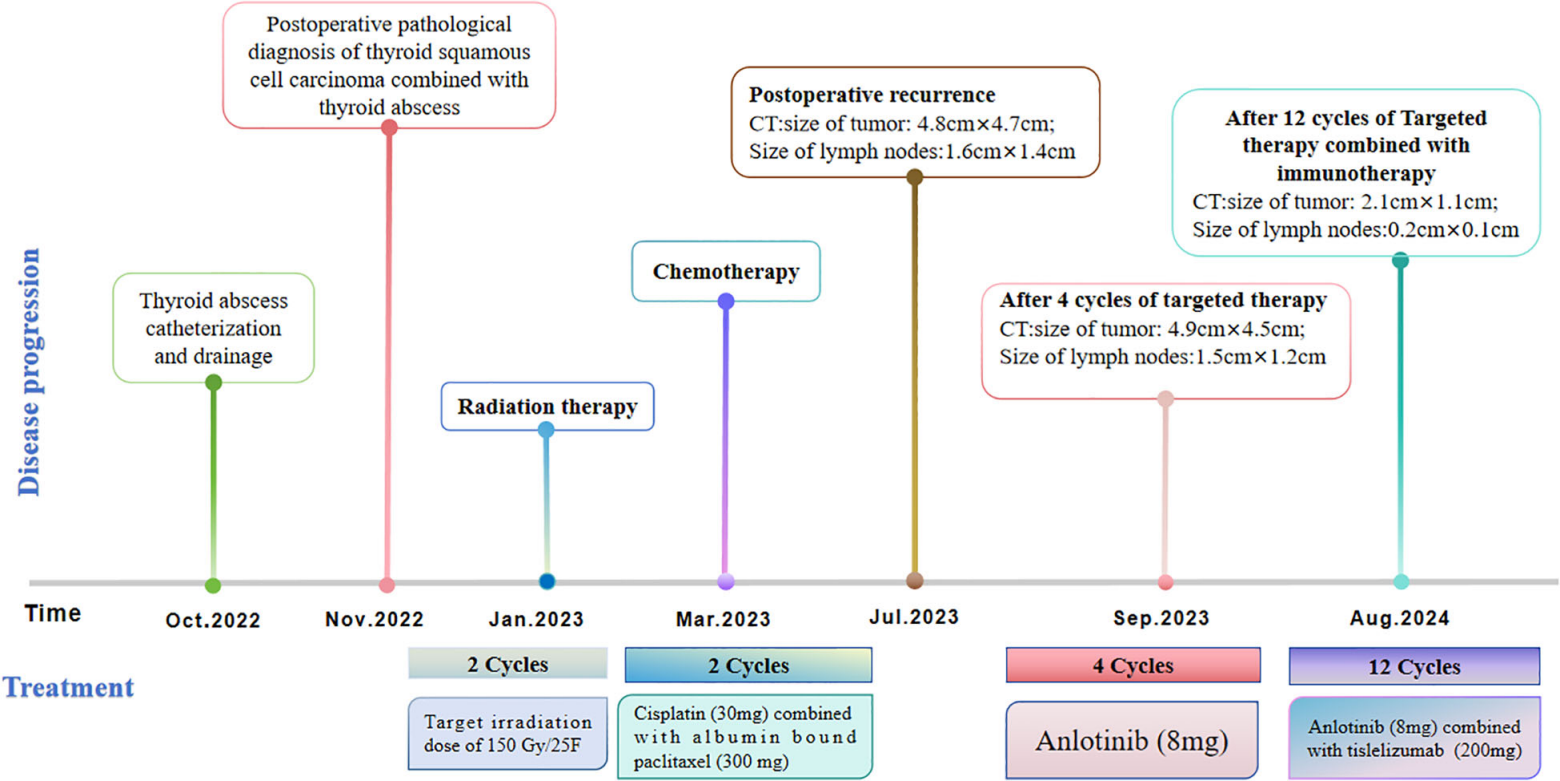
Timeline scheme depicted the major clinical events experienced by the patient since initial diagnosis.

Considering the limitations of existing therapeutic options and the current complexities in treatment decisions, managing this patient’s postoperative recurrence presents significant challenges. The multidisciplinary team (MDT) has recommended the administration of a multi-targeted tyrosine kinase inhibitor, tailored to her specific clinical condition. She commenced oral administration of anlotinib at a dosage of 8 mg per day, with each cycle lasting 21 days, starting on July 4, 2023. Upon review of a neck-enhanced CT scan after four cycles, the lesion size remained relatively unchanged at 4.9cm×4.5cm (**Table 2**). Given the promising potential of combining targeted therapy with immunotherapy for the treatment of solid tumors (8, 9), we decided to initiate a treatment regimen combining anlotinib with tislelizumab. Beginning on September 26, 2023, a treatment regimen consisting of anlotinib 8 mg (administered in 21-day cycles) combined with tislelizumab 200 mg (also in 21-day cycles) was initiated. The reduction of the recurrent neck mass was assessed through CT imaging at the completion of the 4th, 6th, 8th, and 12th cycles, respectively (**Table 2**). Follow-up computed tomography(CT) scans indicated a continuous reduction in the neck mass, with no detectable left cervical and submandibular lymph nodes. The treatment efficacy was evaluated as a partial response (PR). Subsequent circulating tumor cell (CTC) tests revealed a significant decrease in single-cell CTCs compared to previous measurements (**Table 1**). After 12 months of combination therapy, there was a significant reduction in the patient’s recurrent tumor and metastatic lymph nodes, effectively inhibiting tumor progression. The patient underwent a total thyroidectomy, effectively excising all thyroid tissue potentially involved in Hashimoto’s thyroiditis. The diagnosis of Hashimoto’s thyroiditis did not alter our subsequent treatment decisions. Postoperatively, we continued to monitor the patient’s thyroid function and administered thyroid hormone replacement therapy as needed. However, during the treatment period, the patient experienced discomforting symptoms, including grade III skin damage, grade IV myelosuppression, neurotoxic reactions, and pronounced fatigue. Given the significant response to the combination regimen of anlotinib and tislelizumab, the survival benefit outweighed the short-term toxicities. Consequently, the targeted combination immunotherapy was continued following aggressive symptomatic management of the adverse effects encountered during the treatment period. Currently, the patient is able to engage in some daily activities with light physical exertion, as indicated by an ECOG performance status score of 1, and she remains under active treatment. The entire treatment process of the patient from initial diagnosis with ATC-PSCCT to the present (**Figure 4**).

**Table 2:** Comparative enhanced CT of the neck illustrating lesion size pre- and post-treatment.

	Neck Enhanced CT			
	Post relapse lesions	Size of tumor lesion(cm)	Left submandibular lymph node	Size of lymph nodes(cm)
Post-relapse Pre-treatment	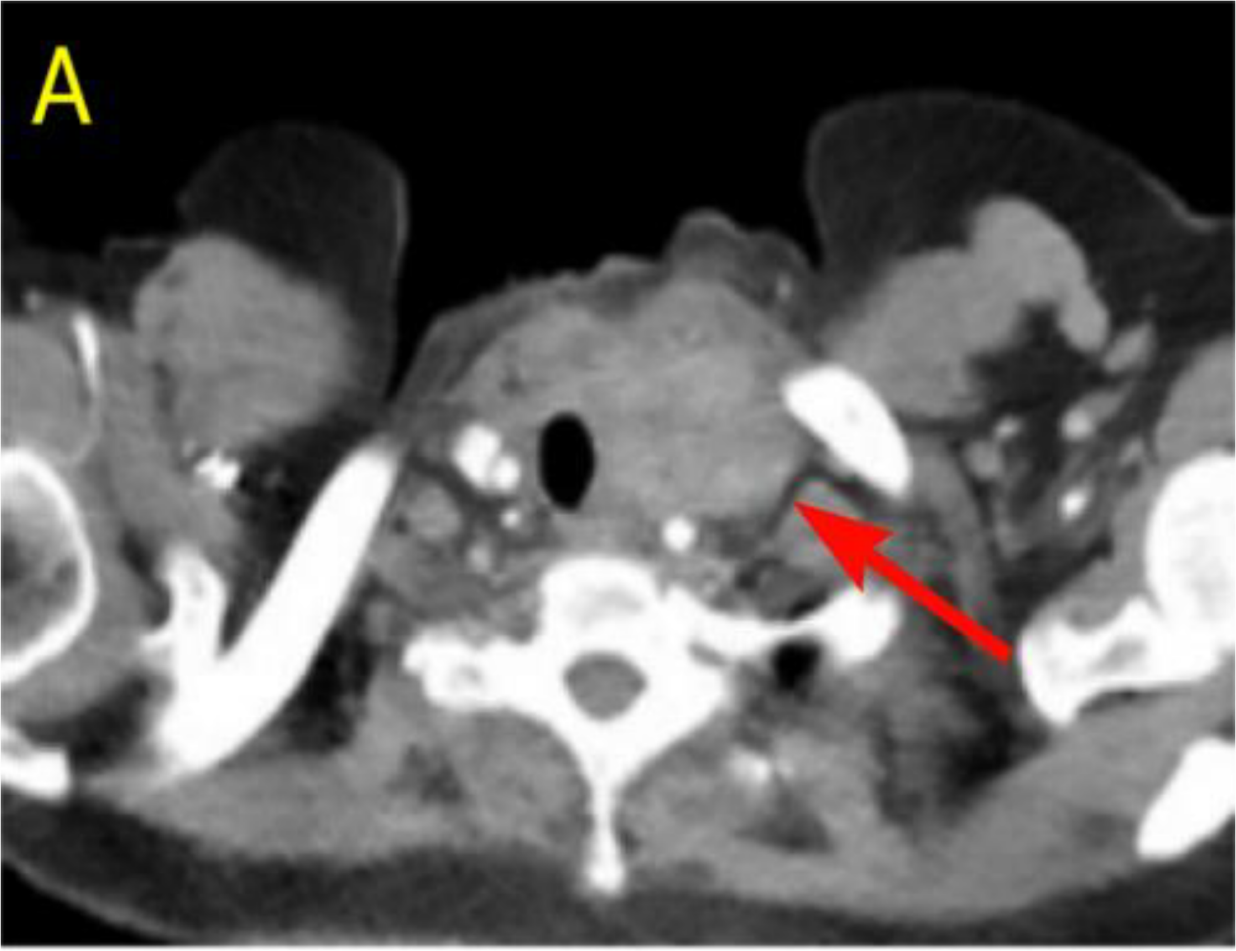	4.8×4.7	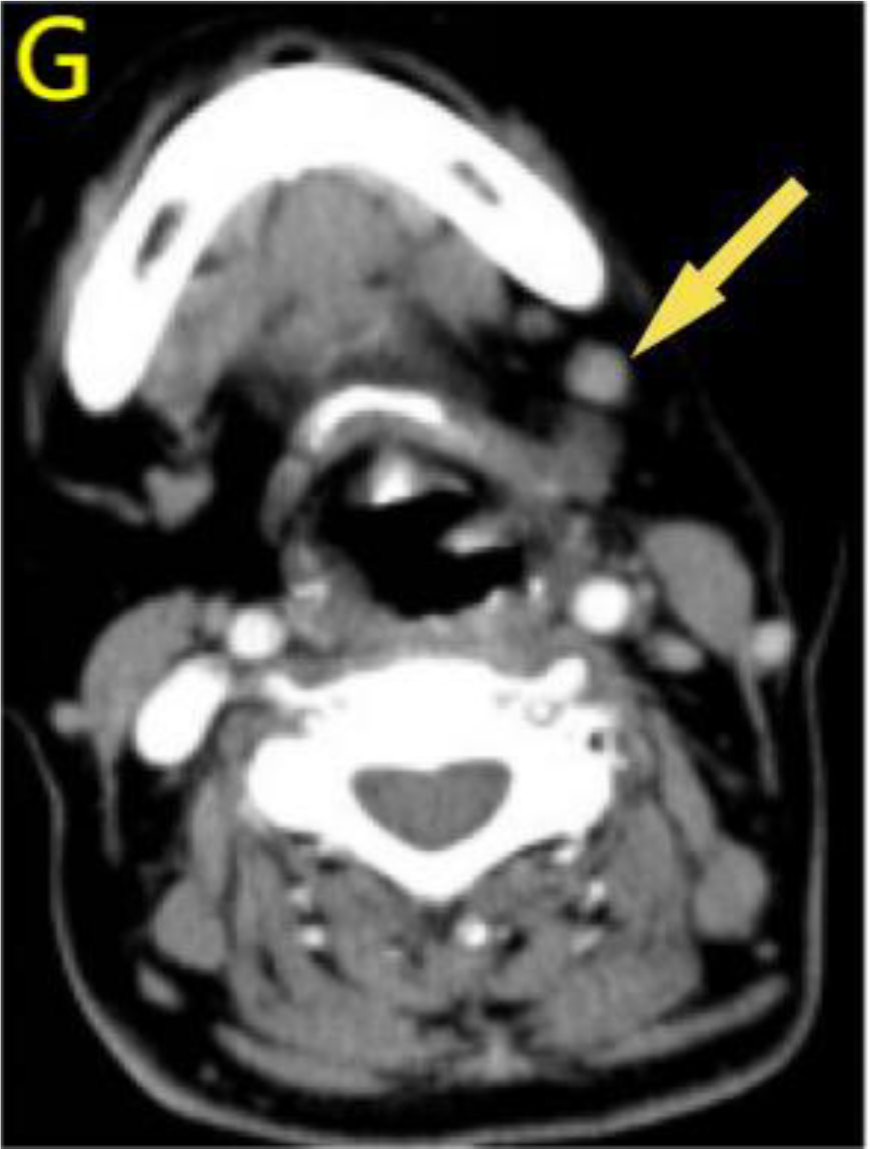	1.6×1.4
After 4 cycles of anlotinib treatment	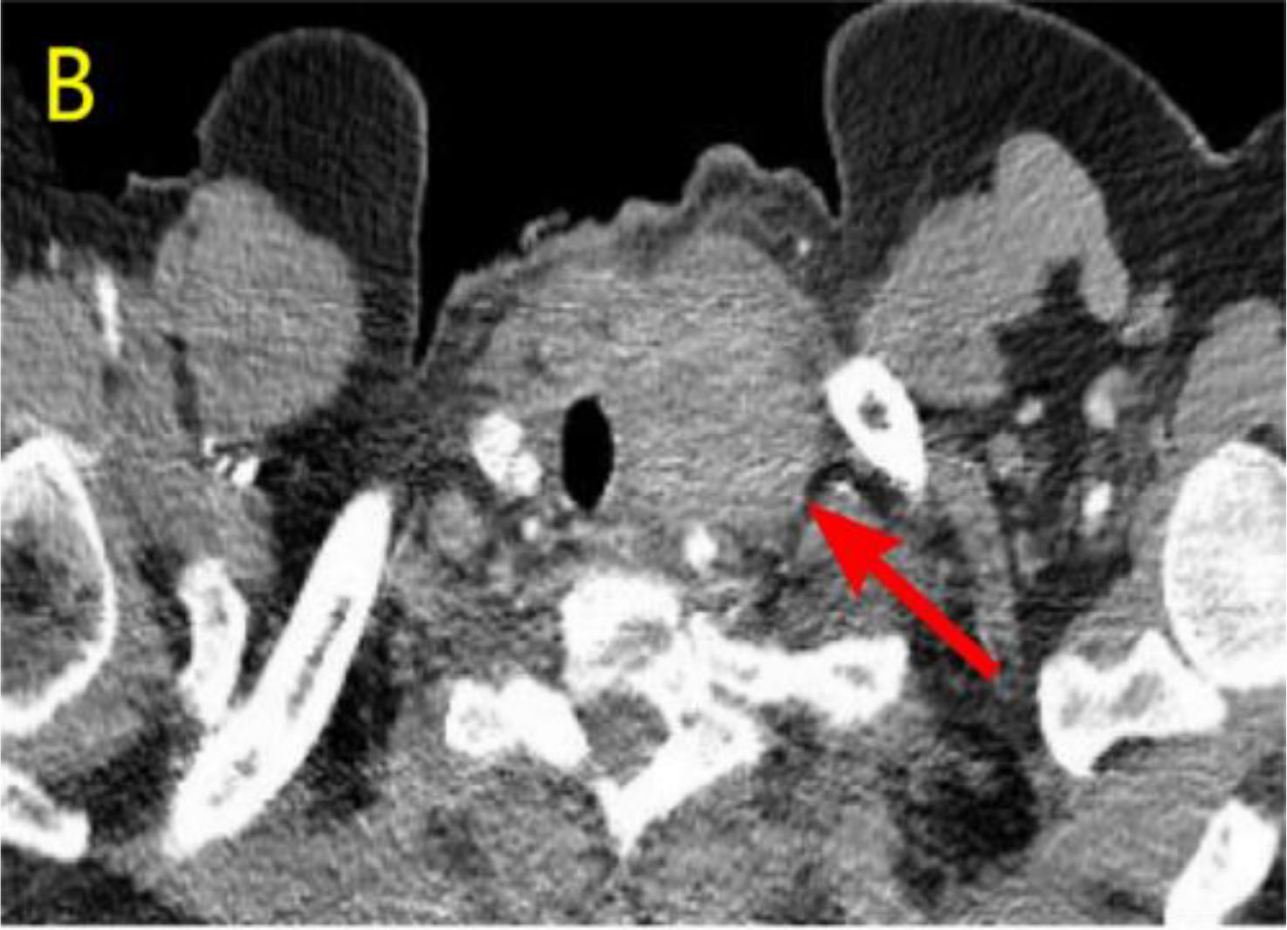	4.9×4.5	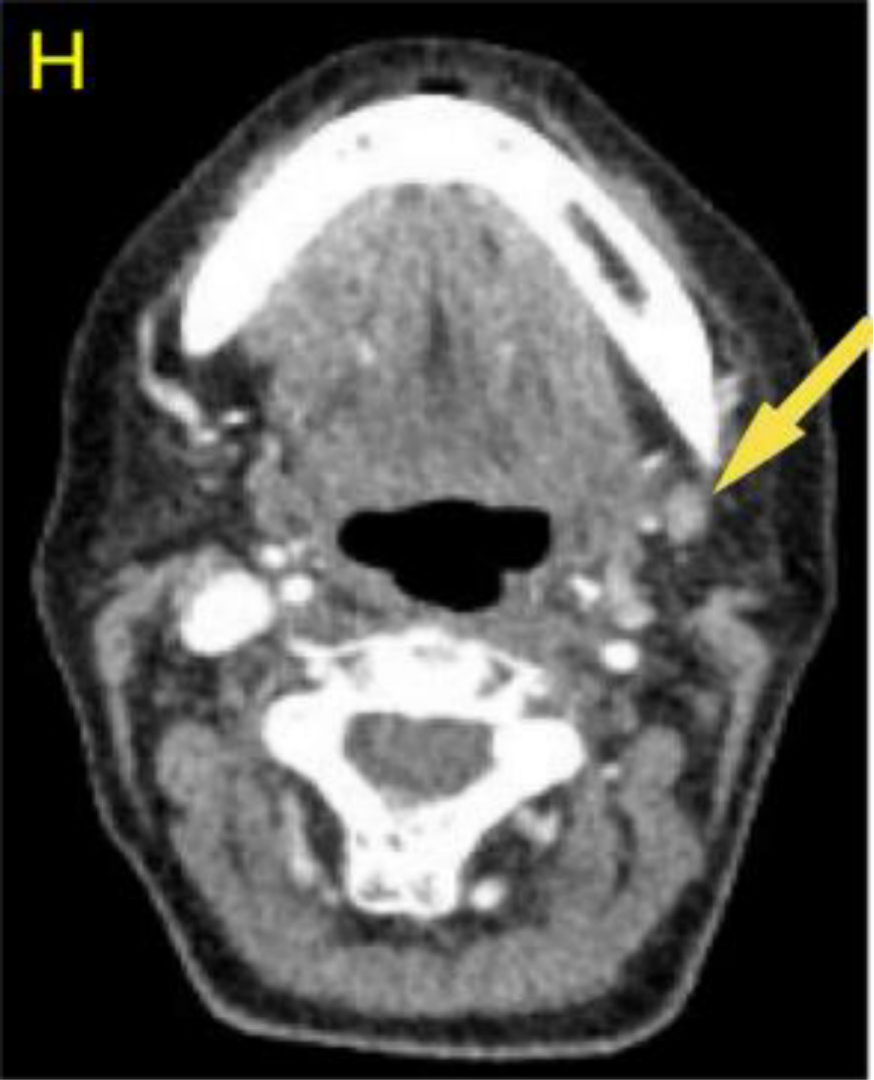	1.5×1.2
After 4 cycles of combination treatment	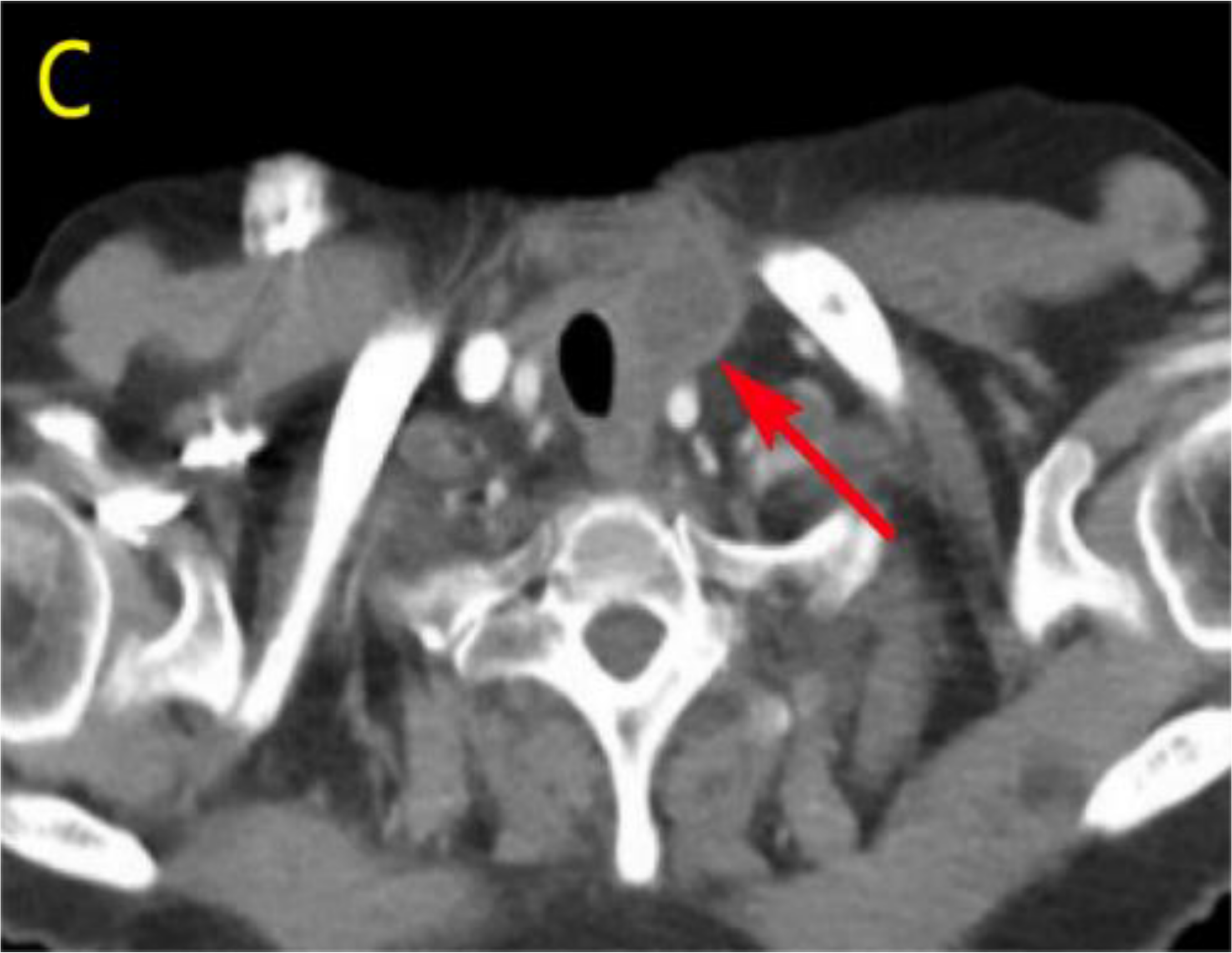	2.9×2.4	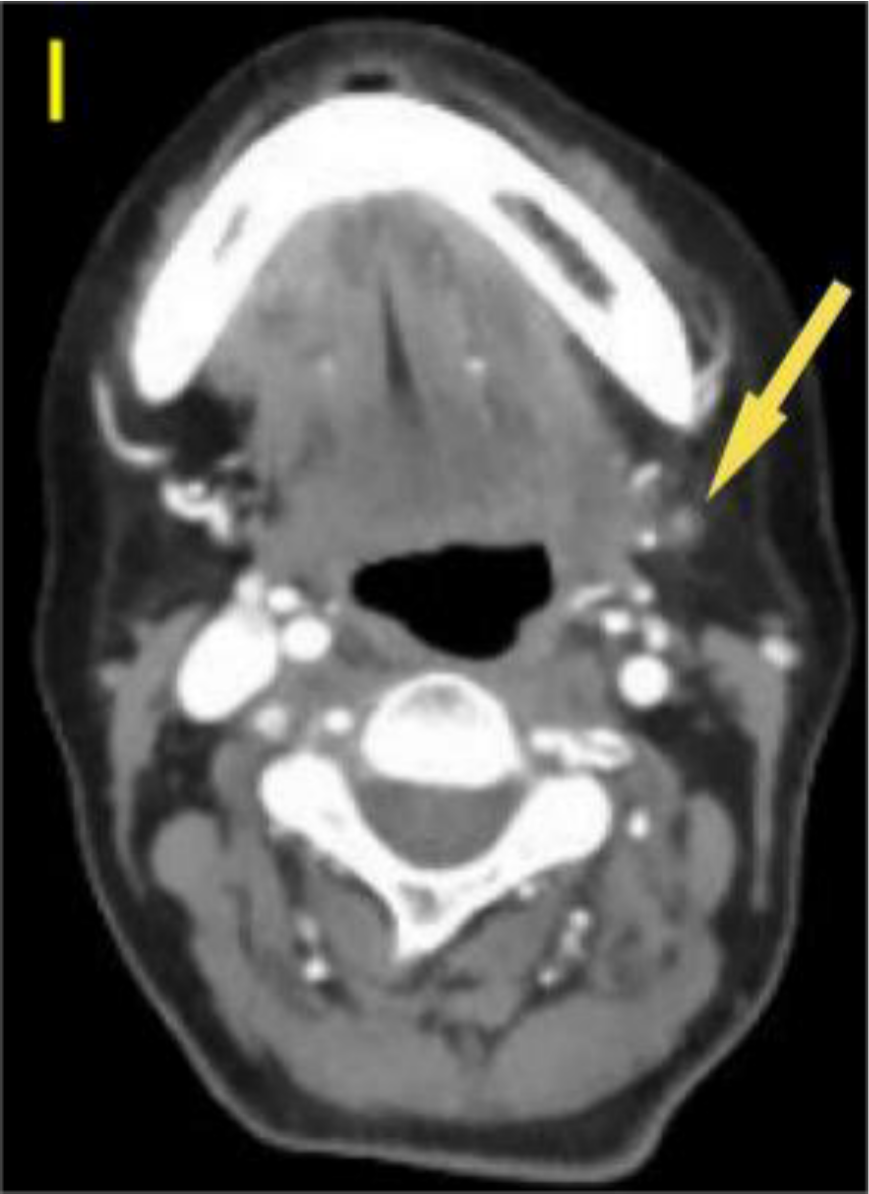	0.8×0.7
After 6 cycles of combination treatment	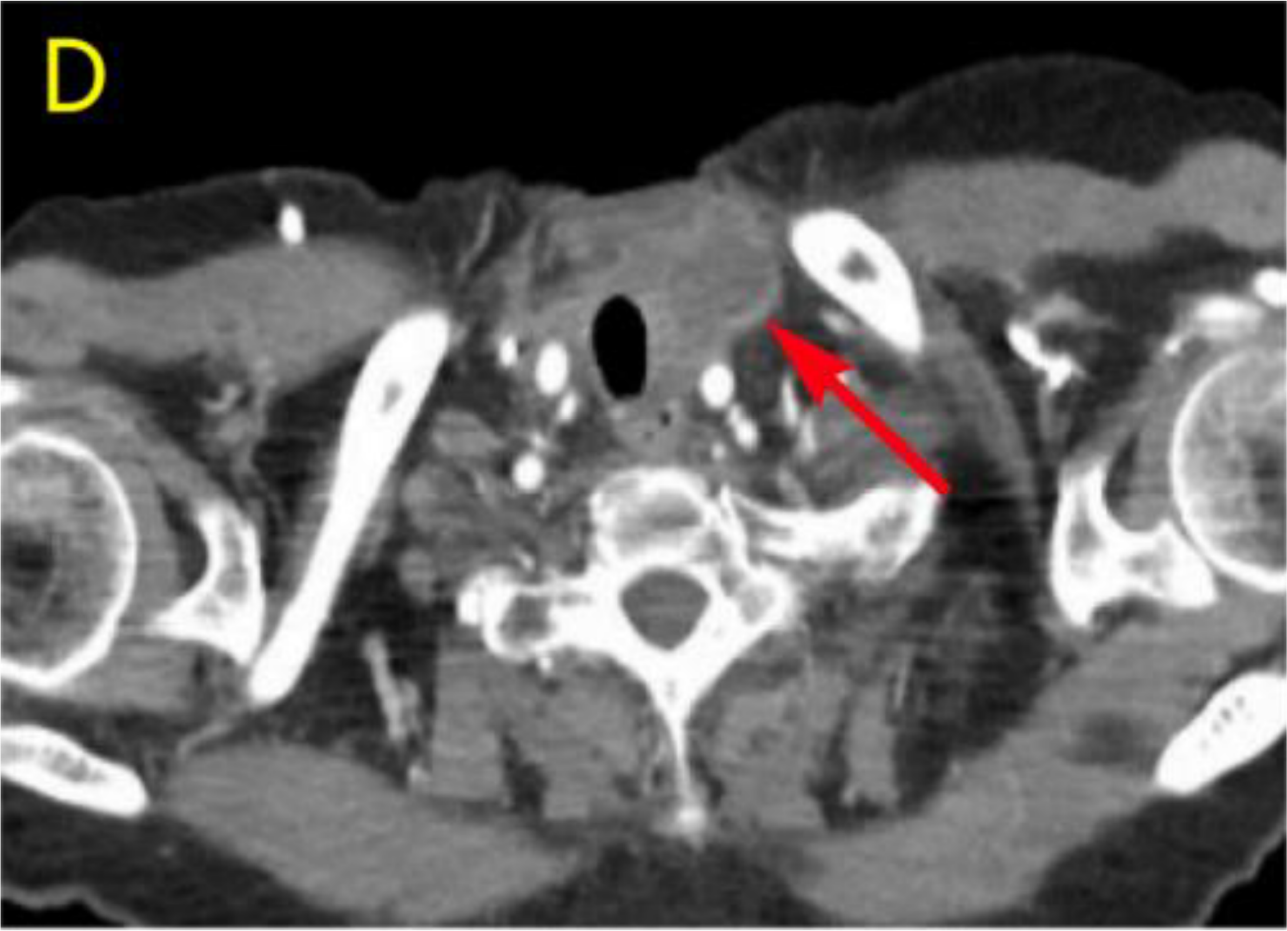	2.7×2.1	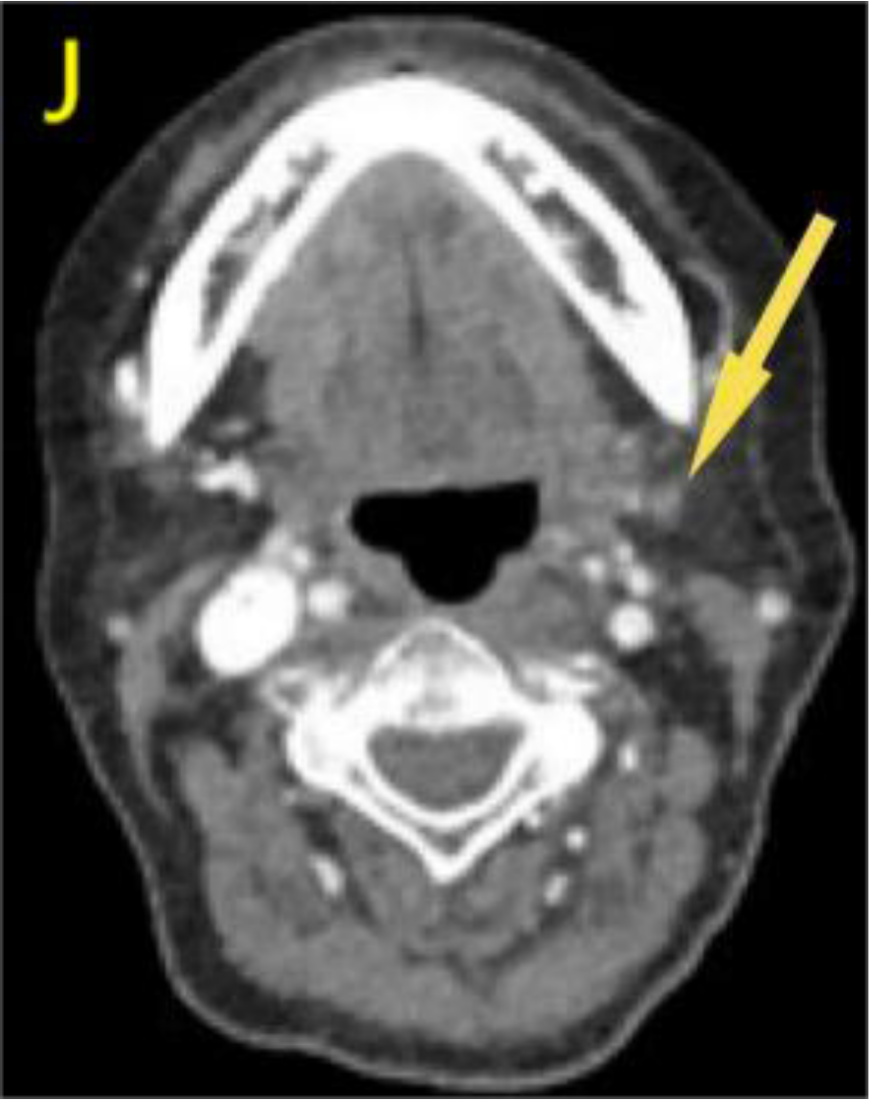	0.5×0.4
After 8 cycles of combination treatment	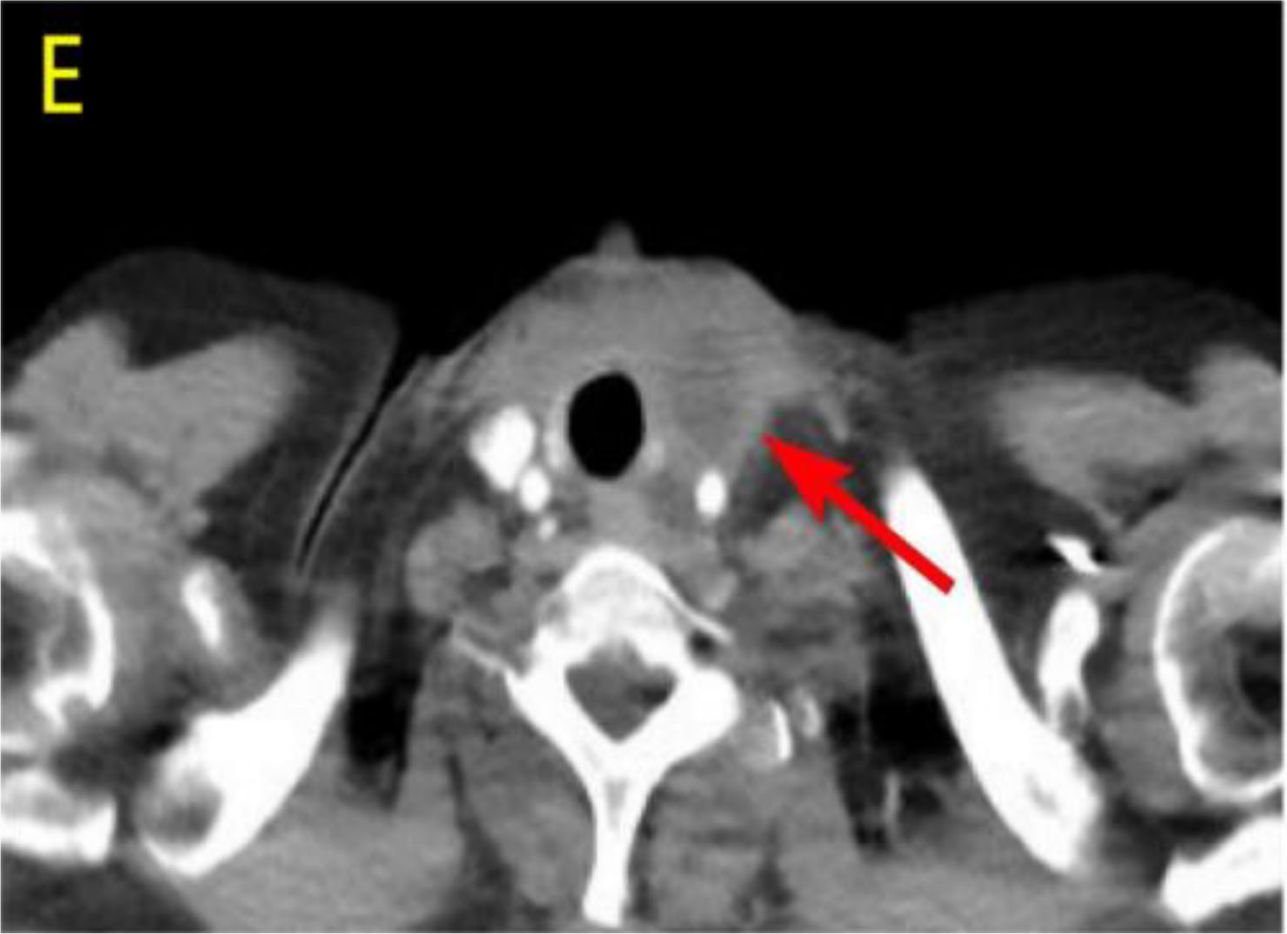	2.3×1.5	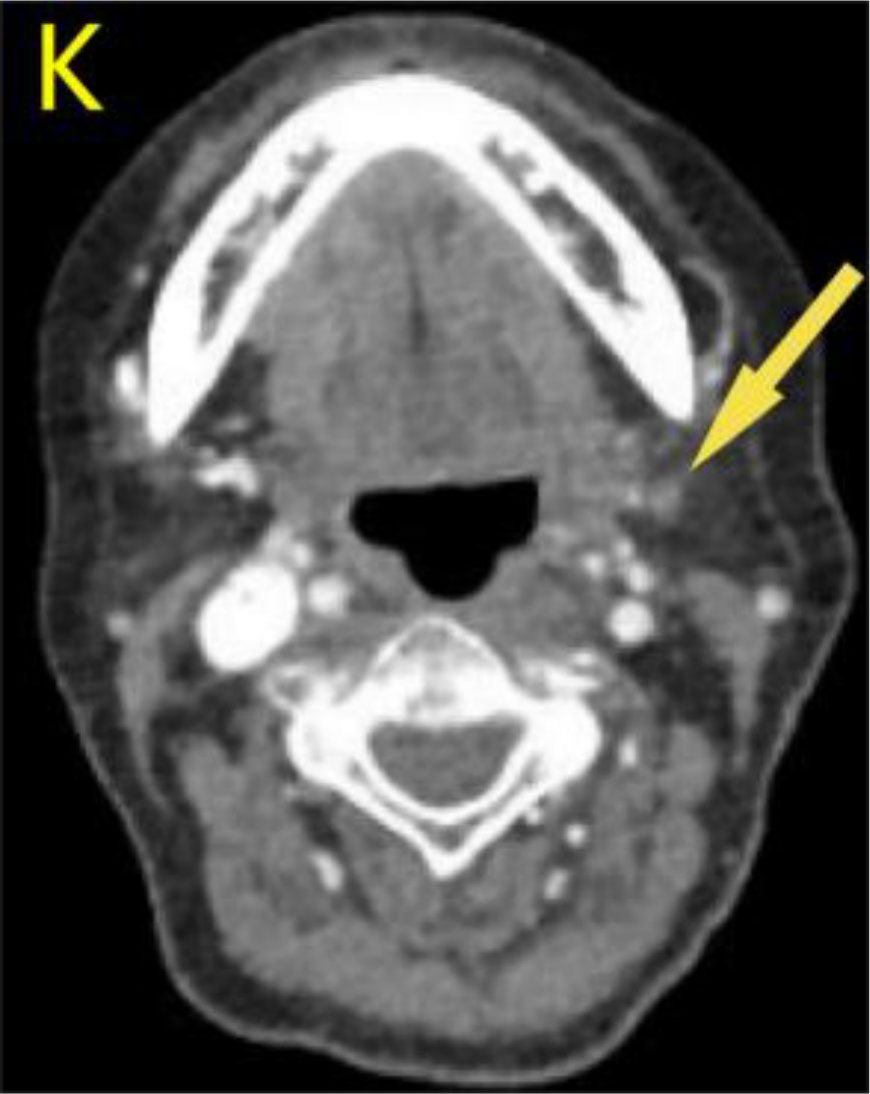	0.4×0.4
**After 12 cycles of combination treatment**	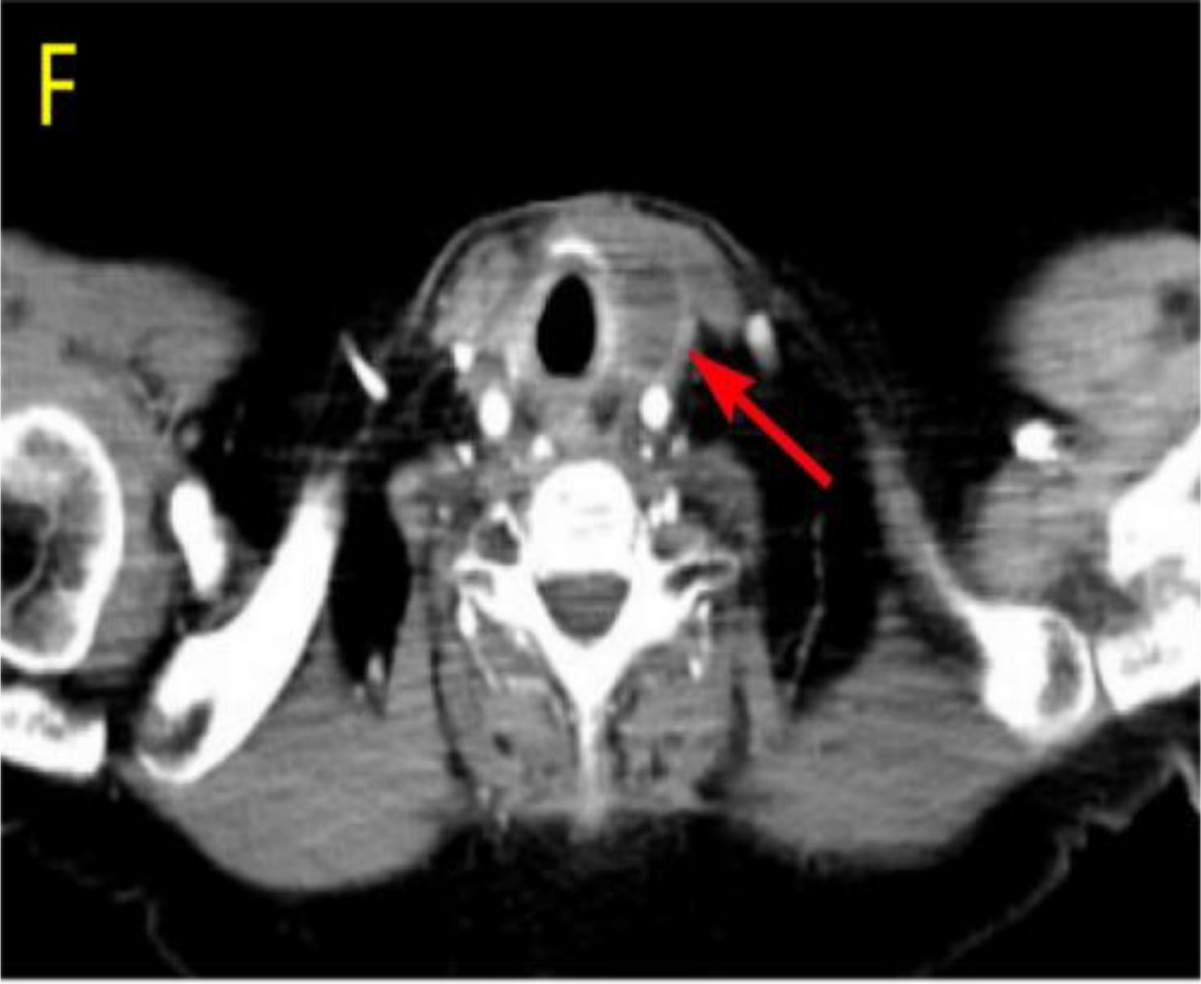	2.1×1.1	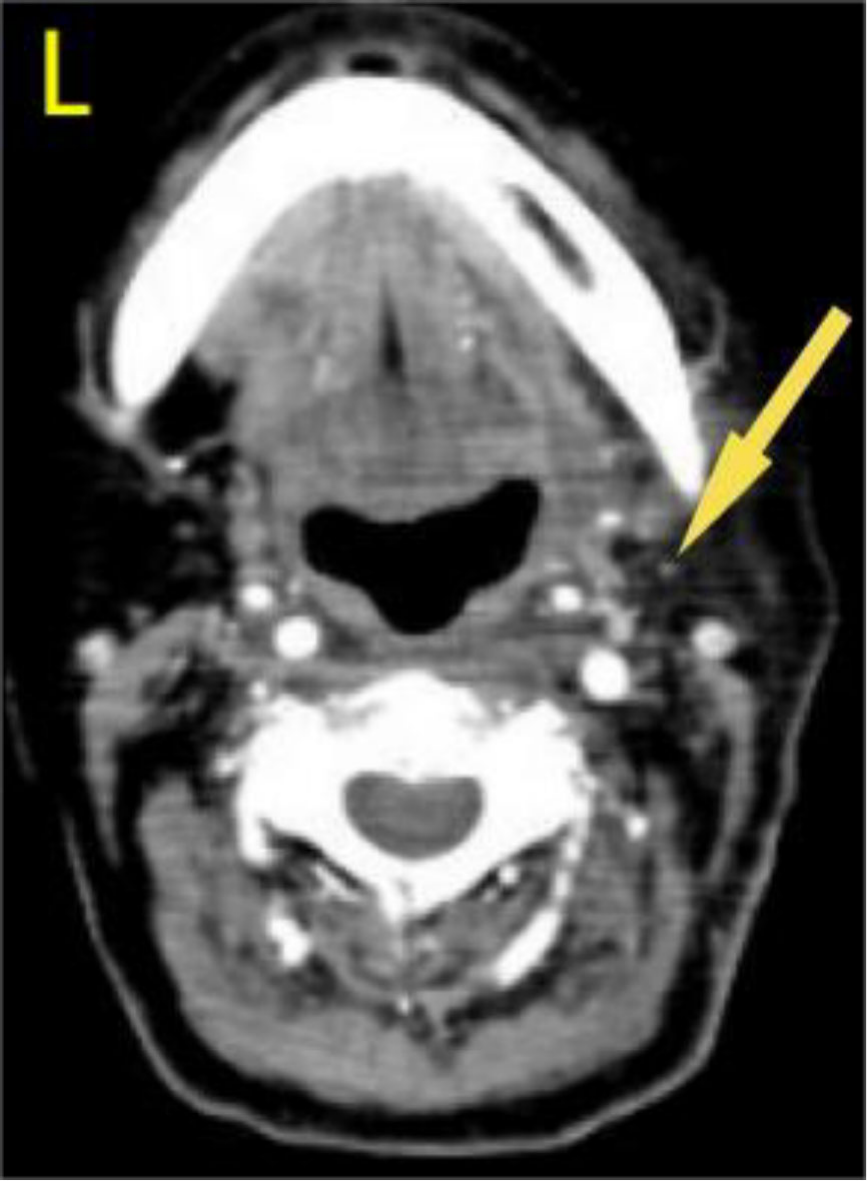	0.2×0.1

## 3 Discussion

### 3.1 The pathogenesis of ATC-PSCCT

ATC is an exceptionally aggressive thyroid malignancy characterized by a markedly poor prognosis, with a median survival duration of only 3 to 6 months (10). PSCCT represents an exceedingly rare form of thyroid malignancy. According to the 2022 World Health Organization (WHO) classification of thyroid tumors, PSCCT is categorized as a subtype of undifferentiated carcinoma of the thyroid (2). The pathogenesis of PSCCT is primarily explained by three prevailing theories. One of these, the embryonic residual theory, posits that PSCCT arises from malignant transformations in epithelial cells that persist from embryonic development within the thyroglossal ducts and parotid fissure tissues. The follicular cell chemotaxis theory posits that follicular epithelial cells, or the squamous chemotaxis of the follicular epithelium, may transform into PSCCT under conditions of chronic irritation. Conversely, the dedifferentiation theory suggests that PSCCT may arise from cellular dedifferentiation in benign or malignant thyroid diseases (11–14). Considering the pathological findings in this case, which include ATC combined with a squamous carcinoma component and Hashimoto’s thyroiditis, the follicular chemotaxis theory appears to be a more plausible explanation for the pathogenesis of this case.

### 3.2 Thyroid abscess

Thyroid abscesses are exceedingly uncommon, constituting less than 0.7% of surgical thyroid pathologies (6). These abscesses typically present unilaterally, and their diagnosis is frequently delayed due to nonspecific clinical manifestations. As the condition advances, it can become life-threatening, potentially leading to complications such as thyroid storm and descending necrotizing mediastinitis (15). Conversely, the occurrence of ATC concomitant with an abscess is extraordinarily rare (16). Furthermore, the reduced thickness of the squamous epithelial cell layer and the increased permeability of intercellular tight junctions can elevate the susceptibility to bacterial infections (17). Chronic inflammation in the adjacent tissues may further compromise the local immune response, thereby facilitating the dissemination of infections (18). The management of thyroid abscesses typically involves a combination of antibiotic therapy and local drainage, with surgical intervention being necessary in cases of persistent infection or extensive necrosis. Ilyin et al. (19) recommended needle drainage for small lesions (< 3 cm) and tubular drainage for lesions exceeding 3 cm or involving the thyroid or parotid glands. Given the rarity and complexity of this case, a multidisciplinary team initially opted for tube drainage and antibiotic therapy to alleviate symptoms. Subsequently, the patient’s prognosis was enhanced through a combination of surgical intervention, radiotherapy, and additional treatments.

### 3.3 Treatment

#### 3.3.1 Surgery and radiotherapy

ATC exhibits rapid progression and a high propensity for local invasion and metastasis. Surgical intervention remains a prevalent treatment modality for ATC. According to the American Thyroid Association (ATA) guidelines, patients who undergo surgical resection demonstrate a higher overall survival rate compared to those who do not, particularly in cases of limited lesions (Stage IVA/Stage IVB) and when complete resection of the lesion (R0/R1) is achieved (20). Given the rapid progression of ATC, radiotherapy represents a critical modality for systemic treatment. Paclitaxel, often administered in conjunction with anthracyclines or platinum-based chemotherapeutic agents, is the most prevalent therapeutic option for patients diagnosed with ATC (20). Notably, even after receiving multimodal therapy (surgery, radiotherapy, chemotherapy), most patients still relapse or metastasize within a short period of time. We integrated the current novel targeted therapy and immunotherapy strategies for recurrent or metastatic ATC through literature analysis (**Table 3**) (21–25). The management of PSCCT remains in the investigational stage, with the extant data predominantly derived from case reports and limited-scale studies. Surgical resection continues to be the principal therapeutic approach. Cho et al. (5) conducted a systematic meta-analysis of PSCCT, indicating that complete surgical resection of the tumor was the sole factor influencing patient prognosis. For patients with PSCCT exhibiting peripheral tissue invasion, surgical intervention presents numerous challenges, often precluding complete resection. Consequently, postoperative adjuvant therapy is employed to manage distant tumor metastasis, thereby enhancing patient survival rates (26, 27). Furthermore, several studies have indicated that paclitaxel exhibits therapeutic efficacy in patients diagnosed with papillary thyroid cancer that includes a squamous carcinoma component, and that postoperative adjuvant therapy may effectively manage distant metastases associated with PSCCT (26, 28). The ineffectiveness of 131I and thyroid-stimulating hormone (TSH) suppression therapy, among other treatments, in managing the tumor can be attributed to the lack of thyroid-stimulating hormone receptors and follicular cells in PSCCT cells (29). This patient presented with PSCCT accompanied by cervical lymph node metastasis. Despite the implementation of a comprehensive treatment strategy that included total thyroidectomy, functional left cervical lymph node dissection, and adjuvant radiotherapy and chemotherapy, the patient experienced local recurrence of metastasis eight months postoperatively. Consequently, the formulation of an effective follow-up treatment plan poses a significant challenge.

**Table 3:** Summary of treatment after ATC recurrence or metastasis.

Authors/trial names	Study design/information	Subjects	Intervention	Effects	Follow-up time	Adverse events
Rukiye et al. (21)	Case report	A 52-year-old woman with recurrence 6 months after surgery and positive BRAF V600	Dabrafenib and trametinib	–	Alive, 2 years of follow-up, no significant tumor recurrence or metastasis	N/A
Xing et al. (22)	Case report	A 47-year-old female patient with ATC experienced recurrence 11 years after initial diagnosis and underwent I^131^ therapy.8 years later, she experienced a second recurrence with metastasis to the lungs. BRAF V600 negative. BRAF V600 negative	Radiotherapy; Tislelizumab	PR(Partial response)	Alive,1 year follow-up, no tumor recurrence or metastasis	No adverse reactions
Gui et al. (23)	Case report	A 67-year-old woman with recurrence 1 month postoperatively	Sintilimab and anlotinib	PR(Partial response),	Alive,18.3 months follow-up, no tumor recurrence or metastasis	N/A
Kollipara et al. (24)	Case report	A 62-year-old man presents with cervical lymph node and lung metastases 9 months after surgery. BRAF V600E positive	Chemotherapy; Vemurafenib and nivolumab	CR(Complete remission)	Alive, 20 months follow-up	Progressive joint pain and generalized; paingrade 2 colitis
Dierks et al. (25)	Retrospective	6 patients with metastatic ATC	Lenvatinib and pembrolizumab	4/6 (66%) Complete remission 1/6 (16%) Stable disease 1/6 (16%) Progressive disease	Median PFS: 16.5 months	Hypertension (63%), fatigue (38%), anorexia (50%); oral mucositis (25%)

#### 3.3.2 Targeted therapy

BRAF mutations have been identified in approximately 45%-74% of ATC patients (30), with BRAF inhibitors demonstrating a 94% overall survival rate one year post-surgery for patients harboring these mutations (10). Recent studies indicate that inhibitors targeting NTRK, RET, and ALK mutations have shown potential in enhancing the prognosis of ATC patients with alterations in these specific genes (31–34). In the present case, where local recurrence of metastasis was observed eight months post-surgery, re-operation and radiotherapy were considered inappropriate following a comprehensive evaluation. The lack of mutations or gene rearrangements in critical genes such as BRAF, TP53, TERT, RET, and NTRK presents significant challenges for subsequent therapeutic interventions. Multi-target Tyrosine Kinase Inhibitors (MTKIs) have emerged as a promising approach, as they are capable of concurrently targeting multiple signaling pathways. This capability potentially mitigates tumor cell resistance and addresses the limitations inherent in single-target therapies (35). Furthermore, multitarget kinase inhibitors (MTKIs) possess the capability to concurrently inhibit multiple critical signaling pathways, including RAS/RAF/MEK and PI3K/AKT/mTOR, which are integral to their broad-spectrum antitumor efficacy. Previous research has indicated that MTKIs exhibit significant antitumor activity and favorable safety profiles in the treatment of various malignant tumors (36). Consequently, we contemplated the application of MTKIs as a subsequent therapeutic intervention for this patient. The FDA has approved Lenvatinib for the management of advanced differentiated thyroid cancer following radioactive iodine therapy; however, the efficacy rate of Lenvatinib in ATC patients is limited to 17-24% (37). Anlotinib, a novel oral MTKI, exhibits a broad spectrum of activity by targeting vascular endothelial growth factor receptors 2 and 3 (VEGFR2/3), fibroblast growth factor receptors 1 through 4 (FGFR1-4), platelet-derived growth factor receptors α and β (PDGFRα/β), stem cell factor receptor (c-Kit), and rearranged during transfection (RET) genes. Anlotinib exhibits a manageable safety profile and has demonstrated significant efficacy in the treatment of various solid tumors (38). According to Ruan et al. (39), anlotinib interferes with spindle assembly and arrests the cell cycle at the G2/M phase in thyroid tumor cells, resulting in the inhibition of cell proliferation and the induction of TP53-mediated apoptosis.

Additionally, anlotinib inhibits cell migration by disrupting the formation of actin filaments (F-actin). Hu et al. (40) demonstrated that anlotinib suppresses the growth of head and neck squamous cell carcinoma both *in vivo* and *ex vivo* by inhibiting cell proliferation and promoting apoptosis. Furthermore, anlotinib has been shown to inhibit tumor growth by inducing tumor vascular normalization and remodeling the immune-tumor microenvironment through the modulation of CD4+ T cells (41). These findings provide a theoretical foundation for the personalized, targeted therapy of ATC.

#### 3.3.3 Immunotherapy

Anti-PD-1/PD-L1 immunotherapy has demonstrated significant therapeutic efficacy in patients with locally advanced, unresectable, or metastatic tumors that express PD-1 or PD-L1 (programmed cell death receptor 1 and programmed cell death ligand 1, respectively) (42). However, some studies have indicated that, regardless of PD-1/PD-L1 expression levels, PD-1 inhibitors can benefit patients in immunotherapy (43, 44). Potential mechanisms underlying the response of PD-1/PD-L1 negative tumors to PD-1 inhibitors include challenges in detection techniques, compensatory effects of multi-dimensional biomarkers, and the dynamic interplay of exogenous microbiome interventions with the immune microenvironment (45–48). First, the detection target and spatial heterogeneity may result in insufficient sample representation, potentially missing local characteristics of such tumors. PD-1 is expressed on immune cells rather than tumor cells, and biopsy samples may miss areas with high PD-1 expression (e.g., at the tumor invasion margins) due to sampling bias. Furthermore, PD-1 expression is influenced by factors such as T-cell activation status, cytokines, and treatment interventions (chemotherapy or radiation), meaning that pre-treatment static assessments may not reflect dynamic changes in PD-1 expression (45). Second, several studies have reported that independent PD-1/PD-L1 expression may not be the sole determinant for the application of immune checkpoint inhibitors (ICI). Other high-immunogenicity biomarkers, such as Tumor Mutational Burden (TMB) and Microsatellite Instability (MSI), can also influence immune efficacy. TMB, which quantifies the number of non-synonymous mutations in the tumor genome’s coding regions, reflects the tumor’s neoantigen load. High TMB tumors produce abundant neoantigens, which activate more naïve T-cells, creating an immunogenic microenvironment and thereby enhancing sensitivity to anti-PD-1 immunotherapy (46). The MSI-H/dMMR phenotype is caused by defects in the DNA mismatch repair (MMR) system, leading to high-frequency mutations across the genome, particularly in microsatellite regions. This generates immunogenic neoantigens and creates an “inflammatory microenvironment,” significantly enhancing the efficacy of PD-1 inhibitors (47). Finally, studies have reported that exogenous microbiome interventions that reprogram T-cell functional states can also enhance anti-tumor immune responses. In microsatellite stable colorectal cancer, butyrate produced by *Fusobacterium* inhibits histone deacetylase in CD8+ T-cells, inducing the expression of the TBX21 gene, which suppresses PD-1 expression, alleviates T-cell exhaustion, and thus enhances the efficacy of immunotherapy (48). In summary, even in PD-1 negative tumors, PD-1 inhibitors can exert anti-tumor effects through these mechanisms.

Tislelizumab is a novel humanized IgG4 monoclonal antibody characterized by a high affinity for the PD-1 receptor and a large binding interface, which facilitates a more comprehensive blockade of the PD-1/PD-L1 interaction (49). It has been meticulously engineered to minimize binding to Fcγ receptors and macrophages, thereby preventing T-cell elimination and reducing phagocytosis-related resistance to anti-PD-1 therapy. This approach aids in maintaining T-cell numbers and preserving the antitumor efficacy of the treatment (50, 51). Approved by the National Medical Products Administration (NMPA) in China in December 2019, Tislelizumab is indicated for the treatment of Hodgkin’s lymphoma, non-small cell lung cancer, hepatocellular carcinoma, and other solid tumors under clinical supervision. It currently holds the record for the PD-1 inhibitor with the highest number of approved indications, demonstrating significant efficacy, safety, and broad therapeutic accessibility (52). A 42-year-old female patient with ATC developed recurrence and multifocal lung metastases. After receiving a combination of radiotherapy and toripalimab, the patient’s recurrence and metastatic lesions significantly shrank (22). This suggests that toripalimab has demonstrated significant efficacy in treating this ATC patient, highlighting the advantages of combination therapy. Combination therapy not only enhances anti-tumor effects by directly activating the immune system but also exhibits synergistic effects through targeted therapy.

#### 3.3.4 Combination therapy

In recent years, synergistic antitumor therapies have become a prominent strategy in the treatment of various malignancies. A retrospective analysis demonstrated that the concurrent administration of darafenib and trametinib with pembrolizumab significantly prolonged survival in ATC patients harboring BRAF mutations (53). Another study revealed that the combination of nivolumab and vemurafenib led to a substantial reduction in tumor lesions in ATC patients (24). Furthermore, the combination of sintilimab and anlotinib has demonstrated significant and durable efficacy in the treatment of ATC with postoperative recurrence (23). Given the vascular abnormalities present in certain solid tumors, which contribute to the development of immunosuppression, some patients may not derive benefit from immunotherapy as a monotherapy. Anlotinib inhibits tumor angiogenesis, improves the hypoxic state within tumor tissues, and promotes immune cell infiltration. When combined with PD-1 inhibitors, it demonstrates significant synergistic effects (54). A study conducted by Su et al. (41) indicated that the combination of anlotinib with PD-1 inhibitors mitigated the immunosuppression induced by PD-L1 upregulation following monotherapy. This combination therapy reversed the early depletion of CD4+ T-cells, enhanced the inhibitory effect of PD-1 checkpoints on tumor cells, and improved the overall immunotherapeutic response. Additionally, Liu et al. (55) observed that high expression of PD-L1 on vascular endothelial cells inhibited the infiltration of CD8+ T cells and promoted the accumulation of FoxP3+ T cells within tumor tissues, thereby establishing an immunosuppressive barrier. Simultaneously, anlotinib down-regulated the expression of PD-L1, thereby enhancing the immune microenvironment and overcoming the immunosuppressive barrier, which in turn inhibited tumor growth. Tislelizumab, an immune checkpoint inhibitor, restores T-cell activity and augments antitumor effects by blocking the interaction between PD-1 and PD-L1. Additionally, tislelizumab may facilitate the immune-mediated clearance of tumors through other immune cells, such as natural killer cells and macrophages (56, 57). Therefore, the dynamic regulation of the immune microenvironment and the synergistic effects of combination therapy suggest that PD-1/PD-L1 negativity should not be a criterion for excluding PD-1 inhibitor treatment. Anlotinib combined with PD-1 inhibitors enhances efficacy through different mechanisms, such as modulating the immune microenvironment (41, 54, 55). This synergistic anti-tumor effect provides a new perspective on the use of PD-1 inhibitors in PD-1/PD-L1 negative or low-expression tumors. In this particular case, significant reductions in both the primary tumor lesion and metastatic lymph nodes were observed following 12 cycles of combination therapy. Furthermore, CTC monitoring revealed a marked decrease in mesenchymal CTCs and CTC cell clusters, which are known to exhibit higher metastatic potential and drug resistance compared to other CTC subtypes (58). The patient experienced no notable adverse effects during the combination treatment regimen, except for significant malaise, which was well tolerated. The clinical benefit has persisted for over 20 months, indicating that the treatment regimen of anlotinib combined with tislelizumab demonstrated both efficacy and safety in this patient with ATC with a squamous carcinoma component.

### 3.4 Clinical management and long-term follow-up

The management of postoperative recurrence in ATC-PSCCT presents significant challenges. Recurrent tumors typically demonstrate increased aggressiveness and resistance to pharmacological treatments, necessitating a comprehensive evaluation of various therapeutic strategies. Firstly, meticulous postoperative surveillance is essential for the early identification and intervention of recurrences. Secondly, for recurrent localized lesions, reoperation or localized radiotherapy may be considered to mitigate tumor progression. In this instance of ATC combined with PSCCT and concurrent thyroid abscess, we administered multi-targeted tyrosine kinase inhibitors and immune checkpoint inhibitors following tumor recurrence. This therapeutic approach significantly inhibited tumor progression, resulting in a survival period exceeding 21 months post-diagnosis. These findings indicate that the treatment protocol employed is effective in prolonging patient survival. Immunotherapy has the potential to induce immune-related adverse effects, including skin inflammation and autoimmune diseases, among others, which necessitate vigilant monitoring and appropriate symptomatic management. The clinical management of ATC-PSCCT demands a multidisciplinary approach and tailored treatment strategies. Prognosis can be optimized through the continuous enhancement and refinement of therapeutic protocols, coupled with long-term, close follow-up.

### 3.5 Conclusion

In summary, we present a rare case of ATC-PSCCT characterized primarily by a thyroid abscess. Local recurrence and lymph node metastasis were observed eight months post-radical surgery. Following 12 cycles of combined anlotinib and tislelizumab therapy, the recurrent tumor lesions exhibited significant size reduction, thereby effectively controlling disease progression. The observed efficacy in patients experiencing recurrence following ATC is notably uncommon, indicating that this treatment modality may hold therapeutic potential for individuals with ATC and concurrent postoperative recurrence of the squamous carcinoma component. This warrants further rigorous investigation. Moreover, thyroid abscess, a rare infectious condition, necessitates thorough diagnostic evaluations to elucidate its underlying etiology. Clinicians should heighten their vigilance for potential thyroid malignancy in patients presenting with thyroid abscesses and enhance the diagnostic protocols and examinations to facilitate early detection and intervention.

## Data Availability

The original contributions presented in the study are included in the article/supplementary material. Further inquiries can be directed to the corresponding authors.

## References

[1] O’Neill JP Shaha AR. Anaplastic thyroid cancer. Oral Oncol. (2013) 49:702–6. doi: 10.1016/j.oraloncology.2013.03.440 23583302

[2] Baloch ZW Asa SL Barletta JA Ghossein RA Juhlin CC Jung CK. Overview of the 2022 WHO classification of thyroid neoplasms. Endocr Pathol.2022;. (2022) 33:27–63. doi: 10.1007/s12022-022-09707-3 35288841

[3] Lam KY Lo CY Liu MC. Primary squamous cell carcinoma of the thyroid gland: an entity with aggressive clinical behaviour and distinctive cytokeratin expression profiles. Histopathology. (2001) 39:279–86. doi: 10.1046/j.1365-2559.2001.01207.x 11532039

[4] Au JK Alonso J Kuan EC Arshi A St John MA. Primary squamous cell carcinoma of the thyroid: A population-based analysis. Otolaryngol Head Neck Surg. (2017) 157:25–9. doi: 10.1177/0194599817698436 28397584

[5] Cho JK Woo SH Park J Kim MJ Jeong HS. Primary squamous cell carcinomas in the thyroid gland: an individual participant data meta-analysis. Cancer Med. (2014) 3:1396–403. doi: 10.1002/cam4.2014.3.issue-5 PMC4302690 24995699

[6] Yedla N Pirela D Manzano A Tuda C Lo Presti S. Thyroid abscess: challenges in diagnosis and management. J Investig Med High Impact Case Rep. (2018) 6:2324709618778709. doi: 10.1177/2324709618778709 PMC5971374 29854858

[7] Tarigan TJE Epriliawati M. Thyroid abscess as a clinical manifestation of papillary thyroid carcinoma. Acta Med Indones. (2022) 54:138–41. 35398836

[8] Sun Y Niu W Du F Du C Li S Wang J. Safety, pharmacokinetics, and antitumor properties of anlotinib, an oral multi-target tyrosine kinase inhibitor, in patients with advanced refractory solid tumors. J Hematol Oncol. (2016) 9:105. doi: 10.1186/s13045-016-0332-8 27716285 PMC5051080

[9] Chen DS Hurwitz H. Combinations of bevacizumab with cancer immunotherapy. Cancer J. (2018) 24:193–204. doi: 10.1097/PPO.0000000000000327 30119083

[10] Maniakas A Dadu R Busaidy NL Wang JR Ferrarotto R Lu C. Evaluation of overall survival in patients with anaplastic thyroid carcinoma, 2000-2019. JAMA Oncol. (2020) 6:1397–404. doi: 10.1001/jamaoncol.2020.3362 PMC7411939 32761153

[11] Lévay B Kiss A Oberna F Slezák A Tóth E. A pajzsmirigy primer laphámcarcinomája [Primary squamous cell carcinoma of the thyroid gland]. the thyroid gland]. Orv Hetil. (2023) 164(39):1556–9. doi: 10.1556/650.2023.32858 37778012

[12] Shenoy VS Rao RA Kamath PM Prasad V Haseena S. Primary squamous cell carcinoma of thyroid - A rare Malignant goitre. Indian J Surg Oncol. (2016) 7:467–9. doi: 10.1007/s13193-016-0530-4 PMC5097761 27872538

[13] Lui JT Khalil MN Chandarana SP. Primary squamous cell of the thyroid-an abbreviated clinical presentation. J Otolaryngol Head Neck Surg. (2014) 43:17. doi: 10.1186/1916-0216-43-17 24942336 PMC4094923

[14] Son DH Roh JL Cho KJ. Combined squamous cell carcinoma and follicular carcinoma of the thyroid. Korean J Pathol. (2014) 48:418–22. doi: 10.4132/KoreanJPathol.2014.48.6.418 PMC4284486 25588631

[15] Akdemir Z Karaman E Akdeniz H Alptekin C Arslan H. Giant thyroid abscess related to postpartum Brucella infection. Case Rep Infect Dis. (2015) 2015:646209. doi: 10.1155/2015/646209 25861492 PMC4378610

[16] Jiang X Wang J Deng X Xiong F Zhang S Gong Z. The role of microenvironment in tumor angiogenesis. J Exp Clin Cancer Res. (2020) 39:204. doi: 10.1186/s13046-020-01709-5 32993787 PMC7526376

[17] Osanai M Takasawa A Murata M Sawada N. Claudins in cancer: bench to bedside. Pflugers Arch. (2017) 469:55–67. doi: 10.1007/s00424-016-1877-7 27624415

[18] Crisan D Kastler S Bernhard L Weiss T Scharffetter-Kochanek K Schneider LA. Squamous cell carcinoma mimicking inflammatory cysts or abscesses: Role of high-frequency sonography as supporting diagnostic tool. J Dtsch Dermatol Ges. (2024) 22:580–2. doi: 10.1111/ddg.15333 38402431

[19] Ilyin A Zhelonkina N Severskaya N Romanko S. Nonsurgical management of thyroid abscess with sonographically guided fine needle aspiration. J Clin Ultrasound. (2007) 35:333–47. doi: 10.1002/jcu.20288 17471585

[20] Bible KC Kebebew E Brierley J Brito JP Cabanillas ME Clark TJ Jr. 2021 American thyroid association guidelines for management of patients with anaplastic thyroid cancer. Thyroid. (2021) 31:337–86. doi: 10.1089/thy.2020.0944 PMC8349723 33728999

[21] Arıkan R Telli TA Demircan NC Başoğlu T Ercelep Ö Atasoy BM. Rechallenge with dabrafenib plus trametinib in anaplastic thyroid cancer: A case report and review of literature. Curr Probl Cancer. (2021) 45:100668. doi: 10.1016/j.currproblcancer.2020.100668 33127167

[22] Xing Y Wang Y Wu X. Radiotherapy combined with immunotherapy successfully treated one case of anaplastic thyroid cancer: A case report. Front Oncol. (2023) 13:1125226. doi: 10.3389/fonc.2023.1125226 37256174 PMC10225731

[23] Gui L Liu S Zhang Y Shi Y. A remarkable and durable response to sintilimab and anlotinib in the first-line treatment of an anaplastic thyroid carcinoma without targetable genomic alterations: A case report. Onco Targets Ther. (2021) 14:2741–6. doi: 10.2147/OTT.S305196 PMC8068508 33907417

[24] Kollipara R Schneider B Radovich M Babu S Kiel PJ. Exceptional response with immunotherapy in a patient with anaplastic thyroid cancer. Oncologist. (2017) 22:1149–51. doi: 10.1634/theoncologist.2017-0096 PMC5634777 28778959

[25] Dierks C Seufert J Aumann K Ruf J Klein C Kiefer S. Combination of lenvatinib and pembrolizumab is an effective treatment option for anaplastic and poorly differentiated thyroid carcinoma. Thyroid. (2021) 31:1076–85. doi: 10.1089/thy.2020.0322 PMC8290324 33509020

[26] Del Rosario M Dasanu C Tsai H Johnson R. Primary squamous cell carcinoma of the thyroid with complete response to radical radiotherapy and concurrent cisplatin-based chemotherapy. BMJ Case Rep. (2017) 2017:bcr2016217143. doi: 10.1136/bcr-2016-217143 PMC5256516 28100571

[27] Limberg J Ullmann TM Stefanova D Finnerty BM Beninato T Fahey TJ 3rd. Prognostic characteristics of primary squamous cell carcinoma of the thyroid: A national cancer database analysis. World J Surg. (2020) 44:348–55. doi: 10.1007/s00268-019-05098-5 31399796

[28] Ito Y Higashiyama T Hirokawa M Fukushima M Kihara M Takamura Y. Clinical trial of weekly paclitaxel chemotherapy for papillary thyroid carcinoma with squamous cell carcinoma component. Endocr J. (2012) 59:839–44. doi: 10.1507/endocrj.EJ12-0174 22673602

[29] Dong S Song XS Chen G Liu J. Mixed primary squamous cell carcinoma, follicular carcinoma, and micropapillary carcinoma of the thyroid gland: A case report. Auris Nasus Larynx. (2016) 43:455–9. doi: 10.1016/j.anl.2015.10.011 26589365

[30] Pozdeyev N Gay LM Sokol ES Hartmaier R R Deaver KE Davis S. Genetic analysis of 779 advanced differentiated and anaplastic thyroid cancers. Clin Cancer Res. (2018) 24:3059–68. doi: 10.1158/1078-0432.CCR-18-0373 PMC6030480 29615459

[31] Subbiah V Kreitman RJ Wainberg ZA Cho JY Schellens JHM Sori JC. Dabrafenib and trametinib treatment in patients with locally advanced or metastatic BRAF V600-mutant anaplastic thyroid cancer. J Clin Oncol. (2018) 36:7–13. doi: 10.1200/JCO.2017.73.6785 29072975 PMC5791845

[32] Cocco E Scaltriti M Drilon A. NTRK fusion-positive cancers and TRK inhibitor therapy. Nat Rev Clin Oncol. (2018) 15:731–47. doi: 10.1038/s41571-018-0113-0 PMC6419506 30333516

[33] Offin M Guo R Wu SL. et al.Immunophenotype and response to immunotherapy of RET-rearranged lung cancers. JCO Precis Oncol. (2019) 3:PO.18.00386. doi: 10.1200/po.18.00386 31192313 PMC6561651

[34] Leroy L Bonhomme B Le Moulec S Soubeyran I Italiano A Godbert Y. Remarkable response to ceritinib and brigatinib in an anaplastic lymphoma kinase-rearranged anaplastic thyroid carcinoma previously treated with crizotinib. Thyroid. (2020) 30:343–4. doi: 10.1089/thy.2019.0202 31892283

[35] Falcomatà C Bärthel S Widholz SA Schneeweis C Montero JJ Toska A. Selective multi-kinase inhibition sensitizes mesenchymal pancreatic cancer to immune checkpoint blockade by remodeling the tumor microenvironment. Nat Cancer. (2022) 3:318–36. doi: 10.1038/s43018-021-00326-1 PMC7612546 35122074

[36] Qiao Y Choi JE Tien JC Simko SA Rajendiran T Vo JN. Autophagy inhibition by targeting PIKfyve potentiates response to immune checkpoint blockade in prostate cancer. Nat Cancer. (2021) 2:978–93. doi: 10.1038/s43018-021-00237-1 PMC8562569 34738088

[37] Tahara M Kiyota N Yamazaki T Chayahara N Nakano K Inagaki L. Lenvatinib for anaplastic thyroid cancer. Front Oncol. (2017) 7:25. doi: 10.3389/fonc.2017.00025 28299283 PMC5331066

[38] Shen G Zheng F Ren D Du F Dong Q Wang Z. Anlotinib: a novel multi-targeting tyrosine kinase inhibitor in clinical development. J Hematol Oncol. (2018) 11:120. doi: 10.1186/s13045-018-0664-7 30231931 PMC6146601

[39] Ruan X Shi X Dong Q Yu Y Hou X Song X. Antitumor effects of anlotinib in thyroid cancer. Endocr Relat Cancer. (2019) 26:153–64. doi: 10.1530/ERC-17-0558 PMC6215907 30139768

[40] Hu F Guo L Yu J Dai D Xiong Y He Y. Using patient-derived xenografts to explore the efficacy of treating head-and-neck squamous cell carcinoma with anlotinib. Pathol Oncol Res. (2021) 27:1610008. doi: 10.3389/pore.2021.1610008 34955687 PMC8696349

[41] Su Y Luo B Lu Y Wang D Yan J Zheng J. Anlotinib induces a T cell-inflamed tumor microenvironment by facilitating vessel normalization and enhances the efficacy of PD-1 checkpoint blockade in neuroblastoma. Clin Cancer Res. (2022) 28:793–809. doi: 10.1158/1078-0432.CCR-21-2241 34844980 PMC9377760

[42] Shen X Zhao B. Efficacy of PD-1 or PD-L1 inhibitors and PD-L1 expression status in cancer: meta-analysis. BMJ. (2018) 362:k3529. doi: 10.1136/bmj.k3529 30201790 PMC6129950

[43] El-Khoueiry AB Sangro B Yau T Crocenzi TS Kudo M Hsu C. Nivolumab in patients with advanced hepatocellular carcinoma (CheckMate 040): an open-label, non-comparative, phase 1/2 dose escalation and expansion trial. Lancet. (2017) 389:2492–502. doi: 10.1016/S0140-6736(17)31046-2 PMC7539326 28434648

[44] Shah MA Kojima T Hochhauser D Enzinger P Raimbourg J Hollebecque A. Efficacy and safety of pembrolizumab for heavily pretreated patients with advanced, metastatic adenocarcinoma or squamous cell carcinoma of the esophagus: the phase 2 KEYNOTE-180 study. JAMA Oncol. (2019) 5:546–50. doi: 10.1001/jamaoncol.2018.5441 PMC6459121 30570649

[45] Tumeh PC Harview CL Yearley JH Shintaku IP Taylor EJ Robert L. PD-1 blockade induces responses by inhibiting adaptive immune resistance. Nature. (2014) 515:568–71. doi: 10.1038/nature13954 PMC4246418 25428505

[46] Goodman AM Kato S Bazhenova L Patel SP Frampton GM Miller V. Tumor mutational burden as an independent predictor of response to immunotherapy in diverse cancers. Mol Cancer Ther. (2017) 16:2598–608. doi: 10.1158/1535-7163.MCT-17-0386 PMC5670009 28835386

[47] Ganesh K Stadler ZK Cercek A Mendelsohn RB Shia J Segal NH. Immunotherapy in colorectal cancer: rationale, challenges and potential. Nat Rev Gastroenterol Hepatol. (2019) 16:361–75. doi: 10.1038/s41575-019-0126-x PMC7295073 30886395

[48] Wang X Fang Y Liang W Wong CC Qin H Gao Y. Fusobacterium nucleatum facilitates anti-PD-1 therapy in microsatellite stable colorectal cancer. Cancer Cell. (2024) 42:1729–46. doi: 10.1016/j.ccell.2024.08.019 39303724

[49] Osarogiagbon RU. Tislelizumab-A promising new option for enhancing chemotherapy benefit in treatment for advanced squamous cell lung cancer. JAMA Oncol. (2021) 7:717–9. doi: 10.1001/jamaoncol.2021.0262 33792622

[50] Dahan R Sega E Engelhardt J Selby M Korman AJ Ravetch JV. FcγRs modulate the anti-tumor activity of antibodies targeting the PD-1/PD-L1 axis. Cancer Cell. (2015) 28:285–95. doi: 10.1016/j.ccell.2015.08.004 26373277

[51] Zhang T Song X Xu L Ma J Zhang Y Gong W. The binding of an anti-PD-1 antibody to FcγRI has a profound impact on its biological functions. Cancer Immunol Immunother. (2018) 67:1079–90. doi: 10.1007/s00262-018-2160-x PMC6006217 29687231

[52] Lee A Keam SJ. Tislelizumab: first approval. Drugs. (2020) 80:617–24. doi: 10.1007/s40265-020-01286-z 32185681

[53] Hamidi S Iyer PC Dadu R Gule-Monroe MK Maniakas A Zafereo ME. Checkpoint inhibition in addition to dabrafenib/trametinib for BRAF^V600E^-mutated anaplastic thyroid carcinoma. Thyroid. (2024) 34:336–46. doi: 10.1089/thy.2023.0573 38226606

[54] Li X Wu D Tang J Wu Y. The efficiency and safety of triple-drug combination of albumin-bound paclitaxel, anlotinib and PD-1/L1 inhibitors in the 2^nd^ or above line of advanced NSCLC: A retrospective cohort study. Cancer Manag Res. (2024) 16:1003–12. doi: 10.2147/CMAR.S472196 PMC11318595 39135711

[55] Liu S Qin T Liu Z Wang J Jia Y Feng Y. anlotinib alters tumor immune microenvironment by downregulating PD-L1 expression on vascular endothelial cells. Cell Death Dis. (2020) 11:309. doi: 10.1038/s41419-020-2511-3 32366856 PMC7198575

[56] Chen DS Mellman I. Oncology meets immunology: the cancer-immunity cycle. Immunity. (2013) 39:1–10. doi: 10.1016/j.immuni.2013.07.012 23890059

[57] Ribas A. Adaptive immune resistance: how cancer protects from immune attack. Cancer Discovery. (2015) 5:915–9. doi: 10.1158/2159-8290.CD-15-0563 PMC4560619 26272491

[58] Piñeiro R Martínez-Pena I López-López R. Relevance of CTC clusters in breast cancer metastasis. Adv Exp Med Biol. (2020) 1220:93–115. doi: 10.1007/978-3-030-35805-1_7 32304082

